# Comparative Evaluation of Cytotoxic and Apoptotic Effects of Natural Compounds in SH-SY5Y Neuroblastoma Cells in Relation to Their Physicochemical Properties

**DOI:** 10.3390/molecules30081742

**Published:** 2025-04-13

**Authors:** Antonella Rosa, Federica Pollastro, Valeria Sogos, Franca Piras

**Affiliations:** 1 Department of Biomedical Sciences, University of Cagliari, 09042 Monserrato, Italy; sogos@unica.it (V.S.); fpiras@unica.it (F.P.); 2 Department of Pharmaceutical Sciences, University of Eastern Piedmont “Amedeo Avogadro”, 28100 Novara, Italy; federica.pollastro@uniupo.it

**Keywords:** eupatilin, xanthomicrol, zerumbone, arzanol, SH-SY5Y neuroblastoma, cytotoxicity, apoptosis

## Abstract

The cytotoxic and apoptotic properties of four bioactive natural compounds, the prenylated α-pyronephloroglucinol heterodimer arzanol (ARZ), the methoxylated flavones eupatilin (EUP) and xanthomicrol (XAN), and the sesquiterpene zerumbone (ZER), were compared in SH-SY5Y human neuroblastoma cells to assess their potential as neuroblastoma-specific therapeutics. EUP, XAN, and ZER (2.5–100 μM) exerted marked significant cytotoxicity (MTT assay) and morphological changes after 24 h of incubation, following the order XAN > ZER > EUP > ARZ (no toxic effect). The propidium iodide fluorescence assay (PI, red fluorescence) and NucView^®^ 488 assay (NV, green fluorescence) evidenced a significant increase in the apoptotic cell number, vs. controls, in SH-SY5Y cells pre-incubated for 2 h with the compounds, in the following order of apoptotic potency: XAN > EUP > ZER > ARZ. The PubChem database and freely accessible web tools SwissADME, pkCSM-pharmacokinetics, and SwissTargetPrediction were used to assess the physicochemical/pharmacokinetic properties and potential protein targets of the compounds. At 50 μM, a positive correlation (r = 0.917) between values of % viability reduction and % human intestinal absorption (bioavailability) was observed, indicating a marked contribution of compound membrane permeability to cytotoxicity in SH-SY5Y cells. The capacity of compounds to induce apoptosis emerged as inversely correlated to the computed lipophilicity (r = −0.885).

## 1. Introduction

Plant-derived natural products (phytochemicals) and their derivatives represent a significant source of new therapeutic drugs for cancer therapy [1,2,3]. These compounds can enhance treatment efficacy and reduce adverse reactions in cancer patients [2]. Several natural products are currently being investigated as potential cytotoxic agents and have shown a positive trend in preclinical research [1]. Some typical examples include taxol analogs, vinca alkaloids (vincristine and vinblastine), and podophyllotoxin analogs [1,2,3]. 

**Figure 1:**
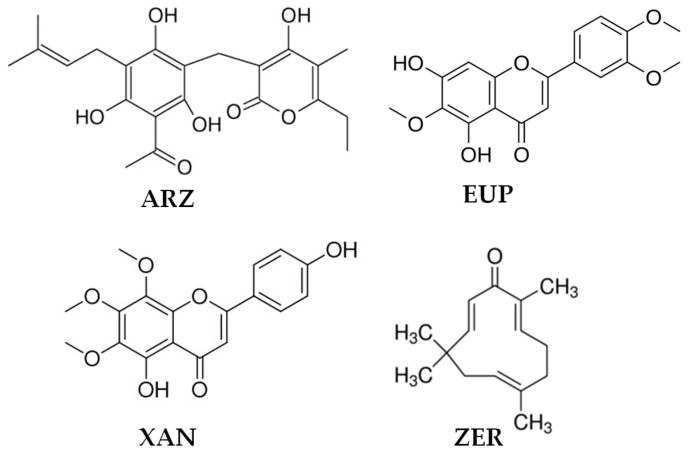
Chemical structures of arzanol (ARZ), eupatilin (EUP), xanthomicrol (XAN), and zerumbone (ZER).

The prenylated pyrone–phloroglucinol heterodimer arzanol (ARZ, Figure 1) is a natural compound isolated from the aerial parts (leaves and flowerheads) of the *Helichrysum italicum* ssp. *microphyllum* [4,5,6] and inflorescences of *Helichrysum stoechas* [7].

This secondary metabolite with a scaffold characterized by a three hydroxylated phenol bearing a *C*-prenylation, and an acetyl moiety united through a methylene bridge with an enolic hydroxyl in the α-pyrone ring, has noteworthy bioactivity, including anti-inflammatory [4,8,9], antioxidant [5,9,10,11,12,13], antimicrobial [4,6], antiviral [4], and anticancer [13,14] effects. This phloroglucinol demonstrated the ability to decrease viability in cancer cells [8,13,14] without cytotoxicity in various normal cells [5,10,11].

Eupatilin (EUP, 5,7-dihydroxy-3′,4′,6-trimethoxyflavone) (Figure 1) is a three-methylated lipophilic flavonoid not ubiquitous in plants and typical of the *Artemisia* species (*A. umbelliformis* Lam., *A. genipi* Weber, and *A. eriantha*) [15,16]. EUP is endowed with a wide range of biological activities, including anti-inflammatory [16], antioxidant [16,17], and anticancer properties [15,16,17,18,19,20,21,22]. This methoxyflavone has been indicated as a potential therapeutic/chemopreventive agent for the treatment of different types of tumors [15]. Inhibition of cancer cell growth/proliferation [15,16,17,18,19,20,21,22], apoptosis induction [15,16,17,18,19,20,21], cell cycle arrest [15,16,22], mitochondria membrane potential reduction [16,17,18], lipid profile modulation [17,20], alteration of cytoskeletal organization [22], prooxidant activity [16,18,20,21], and modulation of cancer cell signaling pathways [15,16,18,19,21,22] are the main mechanisms of EUP antitumor activity.

Xanthomicrol, a three-methylated flavonoid (XAN, 5,4′-dihydroxy-6,7,8-trimethoxyflavone) (Figure 1), has been identified in various traditional medicinal plants, including *Clinopodium douglasii* [23], *Dracocephalum kotschyi* Boiss [23,24,25,26], *Achillea erba-rotta* subsp. *moschata* (Wulfen) I. Richardson (musk yarrow, flowering tops) [20,27], and *Baccharis densiflora* Wedd [28]. XAN has antimicrobial [24], antioxidant [24], anti-inflammatory [24], and anticancer properties [20,23,24,25,26,27,28,29,30]. The in vitro and in vivo antitumor properties of XAN are linked to its ability to induce in cancer cells apoptosis [20,23,26,27,29,30], cell cycle arrest [23,27,29,30], proliferation/viability inhibition [20,23,24,25,26,27,29], lipid profile modulation [20,27], prooxidant effects [20], and modulation of cancer-related enzymes [23,29,30].

The monocyclic sesquiterpene dienone zerumbone (ZER) (2,6,9,9-tetramethyl-[2E,6E,10E]-cycloundeca-2,6,10-trien-1-one) (Figure 1) is a natural dietary compound isolated from the rhizome of *Zingiber zerumbet* Smith (shampoo ginger, an edible ginger) [31,32,33,34,35] and possesses multiple pharmacological properties including antioxidant [31,32,33,34], antipyretic [32], antibacterial [32,33], immunomodulatory [32,33,34], anti-inflammatory [32,33,34], as well as anticancer activities [31,32,33,34,35,36,37,38]. ZER is considered a promising drug for the prevention and treatment of different types of cancer [32,34,38], due to its ability to exert a selective cytotoxic/antiproliferative action towards cancer cells [31,32,33,34,35,36,37,38], apparently related to its covalent interaction with proteins involved in cell proliferation, and a modulation of key apoptotic proteins [32,33,34,35,37,38]. Moreover, cell cycle arrest [37,38], mitochondria membrane potential reduction [35,36,37], lipid profile modulation [35], and prooxidant activity [36,37] are further mechanisms of ZER antitumor activity.

Neuroblastoma is an embryonal extracranial tumor of the peripheral nervous system derived from neural crest cells, mainly affecting children [39,40]. The current treatment for neuroblastoma includes chemotherapy, radiotherapy, surgery, immunotherapy, and gene therapy [39,40,41]. The chemotherapeutic drugs used in the treatment of neuroblastoma include cisplatin, carboplatin, etoposide, cyclophosphamide, doxorubicin, vincristine, and others, which can cause apoptosis by destroying nucleotides or inhibiting mitosis [41]. The main problems of standard chemotherapy are chemoresistance and the risk of organ toxicity [41]. Therefore, there is great interest in identifying novel therapeutic agents for neuroblastoma to reduce adverse side effects and drug resistance [39,40,41]. Various natural extracts and phenolic compounds have shown anticancer properties (antiproliferative effect, apoptosis induction, and cell cycle arrest) on neuroblastoma cells [39].

The SH-SY5Y human neuroblastoma cell line is extensively used in neuroscience research [12,39,40,41,42]. Undifferentiated (neuroblast-like cells) SH-SY5Y cells, characterized by polygonal cell bodies and short processes, express markers of immature neuronal cells, transcriptional regulators, and low levels of dopaminergic markers [12]. A recent study conducted by surface proteomics has evidenced that the undifferentiated SH-SY5Y cell surfaceome significantly overlaps with the human brain and neuronal DRG (dorsal root ganglion neurons) surface proteome, qualifying this cell line as a model to study neuroblastoma-specific therapeutics [42]. 

This study aimed to investigate and compare the anticancer activity of ARZ, EUP, XAN, and ZER on SH-SY5Y cancer cells to explore the potential application of these natural bioactive compounds as a neuroblastoma therapeutic strategy. Moreover, the in silico physicochemical properties and pharmacokinetic/protein target profiles of tested compounds were analyzed and correlated with the observed activity to identify which drug characteristics primarily influence antitumor activity in neuroblastoma cells. The synergy between experimental assessment and computational techniques is essential in the natural drug discovery process for the development of new and effective cancer treatments.

The anticancer properties of compounds were assessed by monitoring cell inhibitory growth effect (MTT assay) and apoptosis/necrosis induction by propidium iodide and NucView^®^ 488 fluorescence assays. The physicochemical/pharmacokinetic properties and the most probable protein targets of the tested compounds were assessed by the PubChem database [43] and the freely accessible web tools SwissADME [44], pkCSM-pharmacokinetics [45], and SwissTargetPrediction [46]. Finally, the bivariate Person analysis was performed to assess the correlations between cytotoxic and apoptotic effects of ARZ, EUP, XAN, and ZER on SH-SY5Y neuroblastoma cells and the compounds’ physicochemical and pharmacokinetic properties. The results of this study provide valuable insights into the potential application of natural anticancer compounds ARZ, EUP, XAN, and ZER in neuroblastoma treatment and highlight structural and physicochemical/pharmacokinetic properties involved in compound activity, useful for the development of novel neuroblastoma therapeutic agents.

## 2. Results

### 2.1. Effect of DMSO on SH-SY5Y Cell Viability (MTT Assay)

**Figure 2:**
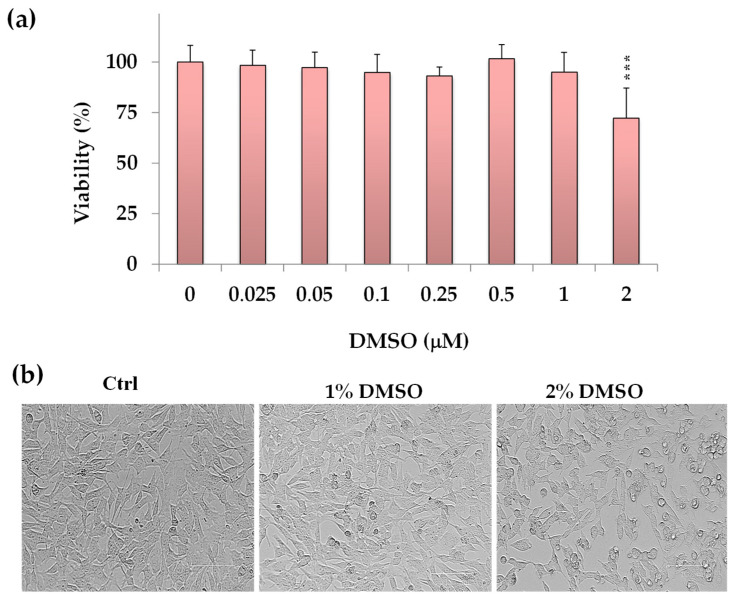
(**a**) Viability, expressed as % of the control (untreated cells, 0), induced by incubation for 24 h with different amounts of DMSO (0.025 to 2% *v*/*v*) in cancer SH-SY5Y cells (MTT assay). Data are presented as mean and standard deviation (SD) of three independent experiments involving six analyses for each sample (n = 18). The statistical significance of differences was assessed by One-way ANOVA, followed by the Bonferroni Multiple Comparisons Test. *** = *p* < 0.001 vs. control cells (0). (**b**) The panels show representative images of phase contrast of control (untreated cells, Ctrl) and cells treated for 24 h with 1% and 2% *v*/*v* of DMSO. Bar = 100 μm.

The colorimetric MTT assay, a method used for evaluating cell viability, proliferation, and cytotoxicity [11,12,13], was employed to assess the impact of DMSO, the solvent used to dissolve the compounds, on cancer SH-SY5Y cell viability. Cell viability data (expressed as a percentage of the control) measured after 24 h incubation with various DMSO concentrations (from 0.025 to 2%) in neuroblastoma SH-SY5Y cells are shown in Figure 2a.

DMSO-exposed neuronal cells showed a viability similar to control cells in the concentration range from 0.25% to 1% *v*/*v*. However, a significant cytotoxic effect (*p* < 0.001 vs. control cells) was observed at a DMSO concentration of 2% *v*/*v*, resulting in a 28% reduction in cell viability. 

Figure 2b displays the corresponding morphological images of control (untreated) cells and cells treated with DMSO (1% and 2% *v*/*v*). At 2% DMSO concentration, decreased cell density areas were observed, and the cells showed a reduction in their cellular size.

Phase contrast microscopy evidenced no significant changes in cell morphology or density in SH-SY5Y cells treated with DMSO at a dose range from 0.25% to 1% *v*/*v* vs. control cells. Therefore, for further experiments, the maximal DMSO concentration to dissolve the compounds was established at 1% *v*/*v* to avoid vehicle toxicity.

### 2.2. Effect of Compounds on SH-SY5Y Cell Viability (MTT Assay)

**Figure 3:**
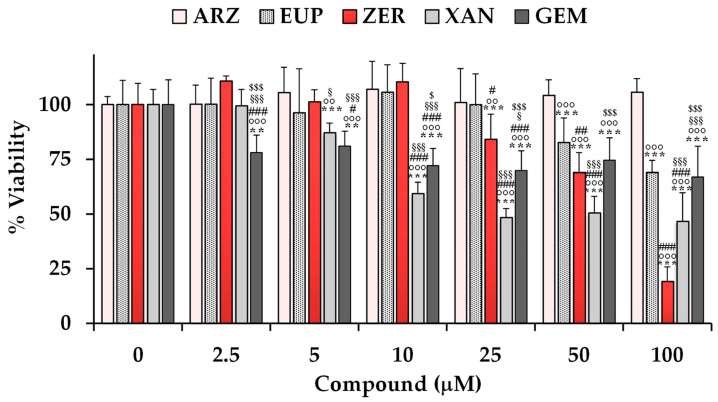
Viability, expressed as % of the control (untreated cells, 0), induced by incubation for 24 h with different amounts (from 2.5 to 100 μM) of arzanol (ARZ), eupatilin (EUP), zerumbone (ZER), and xanthomicrol (XAN), and gemcitabine (positive control, GEM) in cancer SH-SY5Y cells (MTT assay). Data are presented as mean and standard deviation (SD) of three independent experiments involving six analyses for each sample (n = 18). The statistical significance of differences was assessed by one-way ANOVA followed by the Bonferroni Multiple Comparisons Test. For each series, *** = *p* < 0.001, ** = *p* < 0.01 vs. the respective control (0). For each concentration, °°° = *p* < 0.001, °° = *p* < 0.01 vs. ARZ; ^###^ = *p* < 0.001, ^##^ = *p* < 0.01, ^#^ = *p* < 0.05 vs. EUP; ^§§§^ = *p* < 0.001, ^§^ = *p* < 0.05 vs. ZER; ^$$$^ = *p* < 0.001, ^$^ = *p* < 0.05 vs. XAN.

The cytotoxic effects of ARZ, EUP, XAN, and ZER were evaluated on SH-SY5Y neuroblastoma cells using the MTT assay. Figure 3 shows the viability values (expressed as a percentage of the control) measured in control (untreated) cells (100% of viability) and cells treated for 24 h with various concentrations (ranging from 2.5 to 100 μM) of the compounds. Cells were also treated with the same amounts of the anticancer compound gemcitabine (GEM) as a positive control. A previous study evidenced the high sensitivity of various neuroblastoma cell lines to GEM [47].

The treatment with XAN exerted a significant reduction (13%) in cell viability (*p* < 0.001 vs. control cells) at the dose of 5 μM. A significant (*p* < 0.001) cancer cell growth inhibition of 41–53% was observed at the concentration range of 10–100 μM.

ZER, despite a less toxic effect than XAN at low doses, exerted a significant (*p* < 0.001) dose-dependent decrease in cancer SH-SY5Y cell viability in comparison with control cells from the dose of 25 μM, exhibiting a cell growth inhibition of 16–81% at the concentration range from 25 to 100 μM. 

EUP was not significantly toxic at the dose range of 2.5–25 μM, whereas it exerted a significant (*p* < 0.001) reduction in cancer SH-SY5Y cell viability, in comparison with control cells, from the dose of 50 μM, with viability reduction in values of 17% and 31% at the doses of 50 and 100 μM, respectively.

No significant difference in cell viability was observed in SH-SY5Y cells after the treatment with ARZ at all tested concentrations in comparison to the control group. 

The anticancer compound GEM induced a significant decrease in SH-SY5Y cell viability (versus control cells) from the dose of 2.5 μM, exhibiting a cell growth inhibition of 22–33% in the range 25–100 μM. GEN showed lower cytotoxicity than XAN from 10 μM, and similar potency to EUP from 50 μM.

DMSO, used to dissolve the compounds, did not affect cell viability in the range of concentrations used to test compounds (2.5–100 μM, corresponding to 0.025–1% of DMSO). 

Table 1 shows values of IC_50_ (the concentration that decreases the cell viability to 50%) and % viability reduction at 50 μM determined for the compounds in SH-SY5Y cells. 

Cytotoxicity values determined for ARZ [8,11,13,14,35], EUP [15,17,18,20,27,48], ZER [35,37,49], and XAN [20,25,27,28,29] in previous studies in other cancer and normal cell lines are also reported in Table 1 for comparison. 

The IC_50_ values of XAN and ZER after 24 h incubation in neuroblastoma SH-SY5Y cells were 22.8 μM and 69 μM, respectively. In our experimental conditions, it was not possible to determine the IC_50_ value for EUP and ARZ because it exceeded the maximum percentage (1%) of DMSO tolerated in SH-SY5Y cells. 

The values of % viability reduction and the order of potency obtained for XAN, ZER, EUP, and ARZ in SH-SY5Y cancer cells at 50 μM were comparable with those previously obtained for the four compounds in human cervical cancer HeLa cells in similar experimental conditions (24 h of incubation, MTT assay), with XAN showing the highest cytotoxicity (45% viability reduction) [27], followed by ZER (34%) [35], EUP (32%) [17], and ARZ (4%) [13]. Moreover, we previously observed a lower toxic effect of XAN, ZER, and EUP in normal cells than in cancer cells [20,35]. 

**Figure 4:**
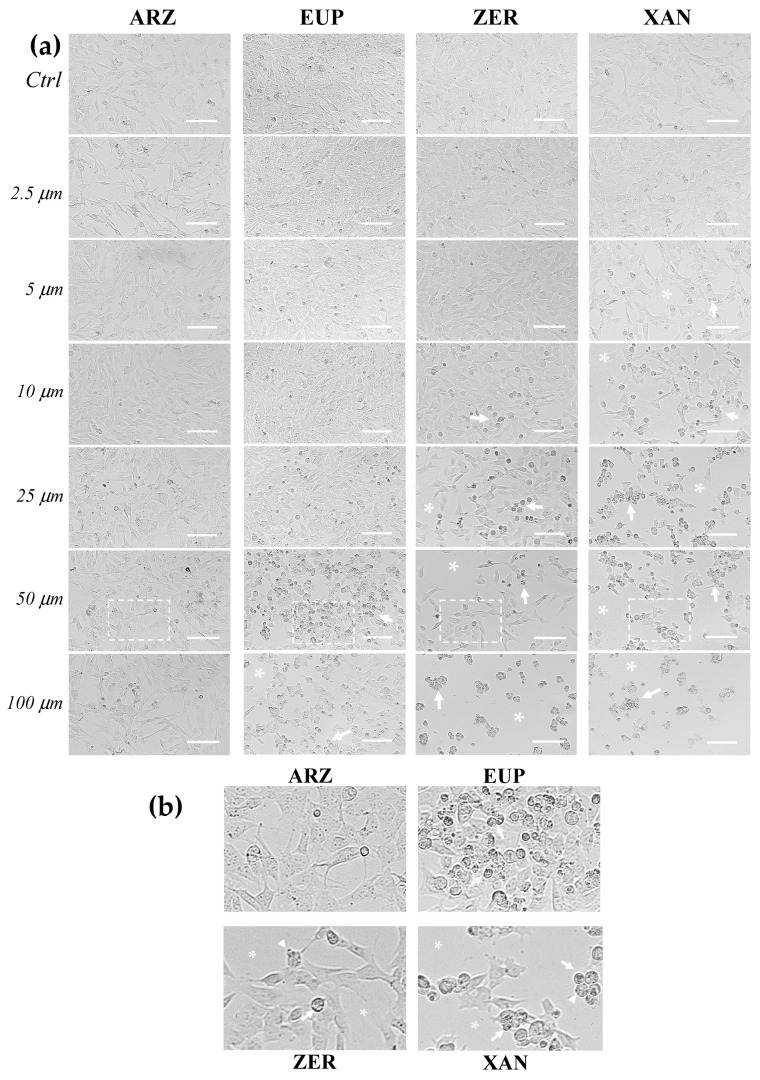
(**a**) Representative phase contrast images of SH-SY5Y control cells (untreated, Ctrl) and cells treated for 24 h with different amounts (2.5 to 100 μM) of arzanol (ARZ), eupatilin (EUP), zerumbone (ZER), and xanthomicrol (XAN). (**b**) Enlargement of images of SH-SY5Y cells treated with the compounds at 50 μM. Asterisks indicate areas characterized by a reduced cell density, while arrows and arrowheads point to rounded/granulated cells and membrane blebbing, respectively. Bar = 100 μm.

Figure 4 shows the representative images observed under phase contrast microscopy of SH-SY5Y control (untreated) cells and cells treated for 24 h with various concentrations (2.5–100 μM) of ARZ, EUP, ZER, XAN, and GEM (positive control).

The microscopic observation of treated cells before the MTT assay allowed us to evidence effects on cancer SH-SY5Y cell morphology induced by 24 h incubation with different compounds. SH-SY5Y control (untreated) cells were fusiform and branched, characterized by short processes. 

The XAN treatment induced a concentration-dependent decrease in the total cell number and an increase in the number of cells characterized by a rounded morphology (apoptotic cells), evident from 5 μM. Moreover, clear apoptotic bodies and cell debris were observed in the dose range of 25–100 μM (Figure 4b). 

Cells with reduced size (rounded cells) were observed in ZER-treated cells from 10 μM, whereas a decrease in the total cell number was monitored from 25 μM.

The microscopic observation of EUP-treated cells evidenced less marked morphology changes than XAN and ZER in the range of 5–25 μM. However, at the highest concentrations (50 and 100 μM), areas with decreased cell density were observed, and the cells displayed changes in size, with a reduced cytoplasm volume and a rounded morphology, clear apoptotic bodies, and cell debris. 

The ARZ treatment did not cause any apparent change in cancer SH-SY5Y cell morphology and number across the range of 2.5–25 μM, and the treated cells were very similar to control cells, as previously observed [12]. However, some cells with altered morphology were evident at 50 and 100 μM, despite no apparent viability reduction. 

The 24 h-treatment with GEM (Figure S1) induced an evident decrease in the number of SH-SY5Y cells with respect to control (untreated) cells. Moreover, cells treated with all GEM concentrations showed an increase in the number of cells characterized by an altered cell morphology (marked size reduction). 

Figure S2 shows the phase contrast images of SH-SY5Y (untreated) control cells and cells treated for 2 h with the highest doses (25, 50, and 100 μM) of ARZ, EUP, ZER, XAN, and GEM. The short time-incubation (2 h) of cancer SH-SY5Y cells with all compounds did not induce a significant viability reduction assessed by MTT assay (<10%). However, microscopic observation after the treatment allowed us to observe different effects of compounds on cell morphology vs. control (untreated) cells.

A decrease in the number of cells with fusiform and branched appearance and an increase in the number of cells characterized by a rounded morphology were especially observed in ZER and XAN-treated cells. Therefore, a short time of incubation was chosen to evaluate and compare the apoptotic effect of compounds. 

The mean values of % viability reduction (% VR) obtained for the four compounds in SH-SY5Y cancer cells at the dose of 50 μM (50%, 31%, 17%, and 0% for XAN, ZER, EUP, and ARZ, respectively) were considered for successive correlation analysis. 

### 2.3. Protective Effect of Compounds Against Apoptosis

The effect of XAN, ZER, EUP, and ARZ on apoptosis and cell death was evaluated by staining cancer SH-SY5Y cells with NucView 488 (NV) dye, an enzyme caspase-3 substrate, capable of detecting the activity of caspase-3/7 within cells (early apoptotic cells) [11,12,20], and with propidium iodide (PI), a DNA-binding fluorescent dye (red) able to evidence late apoptotic and necrotic cells [12]. 

**Figure 5:**
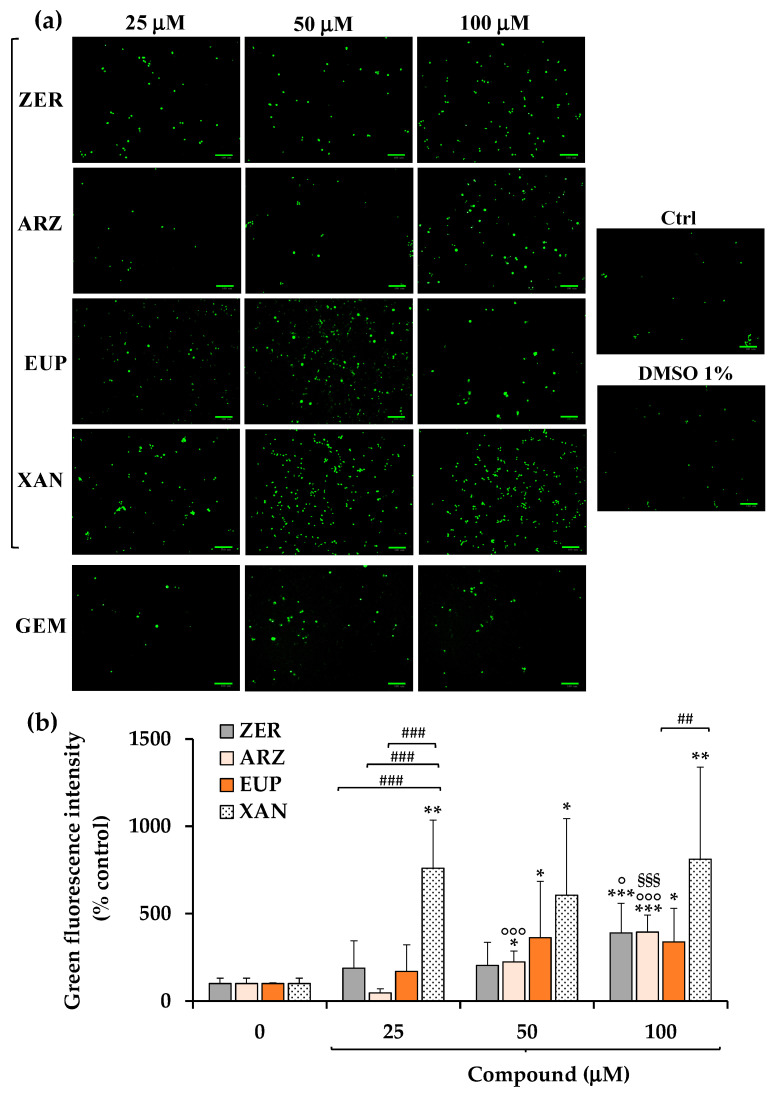
Fluorescence green emission images, by NucView 488 (NV) assay, obtained in SH-SY5Y control (untreated) cells (Ctrl) and cells pre-incubated for 2 h with different concentrations (25, 50, and 100 μM) of zerumbone (ZER), arzanol (ARZ), eupatilin (EUP), xanthomicrol (XAN), gemcitabine (GEM, positive control), and the maximal non-toxic vehicle dose (DMSO 1% *v*/*v*) (**a**). Intensity of NV green emission fluorescence, expressed as % control, obtained by image analysis for cells treated with ZER, ARZ, EUP, and XAN (**b**). All data are presented as mean and standard deviation (SD) of three experiments involving duplicate analyses for each sample (n = 6). The statistical significance of differences was assessed by One-way ANOVA, followed by the Bonferroni Multiple Comparisons Test. For each series: *** = *p* < 0.001, ** = *p* < 0.00, * = *p* < 0.05 vs. the respective control cells (0); °°° = *p* < 0.001, ° = *p* < 0.05 vs. 25 μM; ^§§§^ = *p* < 0.001 vs. 50 μM. For each concentration, between different compounds ^###^ = *p* < 0.001, ^##^ = *p* < 0.01.

Figure 5a shows the green emission images obtained, after 3 h of incubation with NV dye, for SH-SY5Y control (untreated) cells, cells pre-incubated for 2 h with ZER, ARZ, EUP, XAN, and GEM (positive control) (25, 50, and 100 μM), and vehicle treated cells (DMSO 1% *v*/*v*). 

A low basal level of green cells was observed in control (untreated) cells. Cells treated with DMSO, at the maximal non-toxic dose used to dissolve the compounds, showed a green fluorescence emission similar to control cells. An evident increase in green fluorescence was observed in GEM-treated cells at 50 μM, indicating the apoptotic process.

In our experimental conditions, all compounds induced apoptosis in SH-SY5Y, although with a different potency. The quantitative data (expressed as % control) of NV intensity emission fluorescence of ZER, ARZ, EUP, and XAN are reported in Figure 5b.

XAN exhibited the highest pro-apoptotic activity in this model. The incubation of cancer SH-SY5Y cells with XAN induced a significant (*p* < 0.01) rise in the number of rounded and green-fluorescent apoptotic cells in comparison to control cells from the dose of 25 μM (value of 760% of controls), exhibiting comparable values at higher concentrations. An evident induction of apoptosis was observed from 25 μM for EUP (169% vs. control cells) and ZER (188%). 

Despite no apparent viability reduction and low levels of cells with altered morphology observed in cancer SH-SY5Y cells treated for 24 h with ARZ 50 and 100 μM, an evident apoptosis was observed from 50 μM in neuroblastoma cells after 2 h of incubation with the phloroglucinol.

The values of NV intensity emission fluorescence of 389, 394, 338, and 811% were measured at the dose of 100 μM for ZER, ARZ, EUP, and XAN, respectively.

For successive correlation analysis, the mean values of % apoptosis by NV assay (% Apoptosis NV) obtained in SH-SY5Y cancer cells for the four compounds at the doses of 50 μM were considered, corresponding to 605%, 363%, 223%, and 203% for XAN, EUP, ARZ, and ZER, respectively.

**Figure 6:**
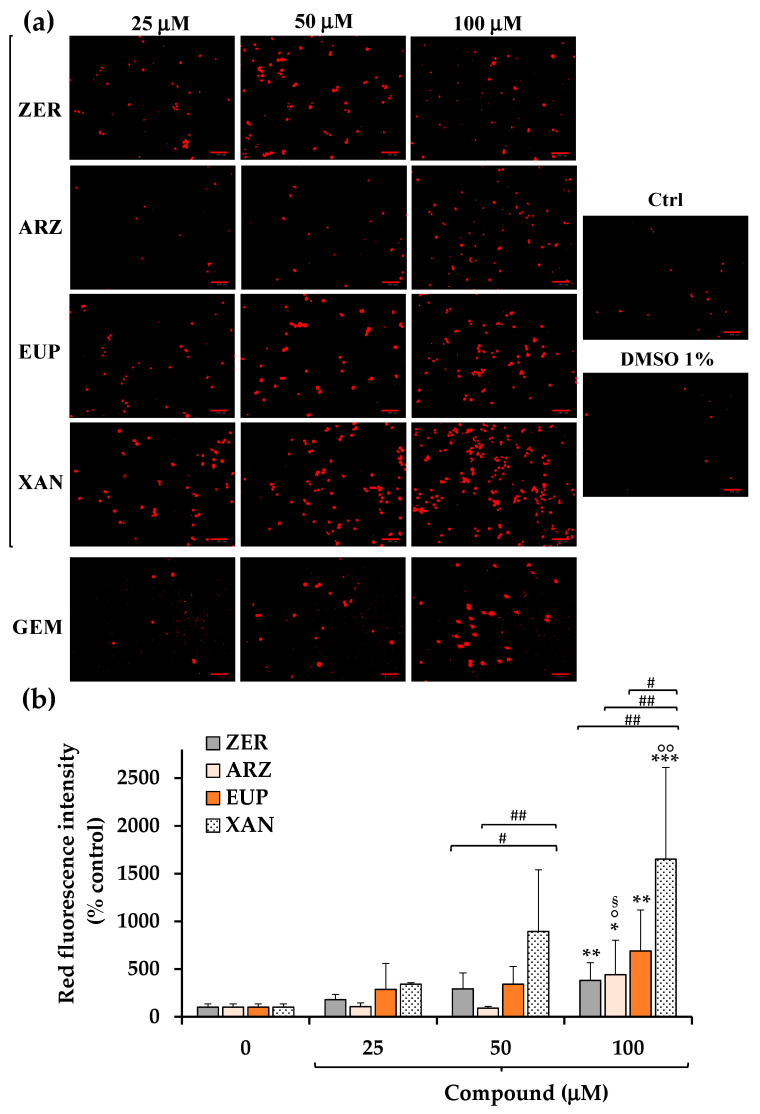
Fluorescence red emission images, by propidium iodide (PI) assay, obtained in SH-SY5Y control (untreated) cells (Ctrl) and cells pre-incubated for 2 h with different concentrations (25, 50, and 100 μM) of zerumbone (ZER), arzanol (ARZ), eupatilin (EUP), and xanthomicrol (XAN), gemcitabine (GEM, positive control), and the maximal non-toxic vehicle dose (DMSO 1% *v*/*v*) (**a**). Intensity of PI red emission fluorescence, expressed as % control, obtained for cells treated with ZER, ARZ, EUP, and XAN by image analysis (**b**). All data are presented as mean and standard deviation (SD) of three experiments involving duplicate analyses for each sample (n = 6). The statistical significance of differences was assessed by One-way ANOVA, followed by the Bonferroni Multiple Comparisons Test. For each series: *** = *p* < 0.001, ** = *p* < 0.01, * = *p* < 0.05 vs. the respective control cells (0); °° = *p* < 0.01, ° = *p* < 0.05 vs. 25 μM; ^§^ = *p* < 0.05 vs. 50 μM. For each concentration: ^##^ = *p* < 0.01, ^#^ = *p* < 0.05 between different compounds.

Figure 6a shows the red emission images obtained, after 2 h of incubation with PI dye, for SH-SY5Y control (untreated) cells, cells pre-incubated for 2 h with ZER, ARZ, EUP, XAN, and GEM (positive control) (25, 50, and 100 μM), and vehicle treated cells (DMSO 1% *v*/*v*). 

A low basal level of red fluorescence was observed in control (untreated) cells. Cells treated with DMSO (1% *v*/*v*) showed a red fluorescence emission similar to control cells. Cells treated with GEM 50 and 100 μM displayed an evident increase in red fluorescence, confirming the apoptotic process, although lower than that observed in EUP- and XAN-treated cells. 

The quantitative data (expressed as % control) of PI intensity emission fluorescence of ZER, ARZ, EUP, and XAN are reported in Figure 6b. 

The 2 h treatment with XAN induced a dose-dependent increase, vs. control cells, in the number of IP-stained cells, with mean values of 343%, 896%, and 1652% of red fluorescence intensity at 25, 50, and 100 μM, respectively, indicating a late apoptosis/necrosis induction. Also, cells treated with ZER and EUP showed a dose-dependent increase in red fluorescence emission, less marked than those induced by XAN at 50 and 100 μM. 

ARZ exhibited a significant increase in the red fluorescence signal in comparison to control cells (*p* < 0.05) only at 100 μM (440%). 

For successive correlation analysis, the mean values of % apoptosis by IP assay (% Apoptosis IP) obtained in SH-SY5Y cancer cells for the four compounds at the doses of 50 μM were considered, corresponding to 896%, 343%, 294%, and 90% for XAN, EUP, ZER, and ARZ, respectively.

### 2.4. In Silico Evaluation of Physicochemical and Pharmacokinetic Properties of ARZ, EUP, XAN, and ZER

In silico evaluations of compound physicochemical, pharmacokinetic, and pharmacological/protein target properties were performed using several web tools, including the PubChem web database [43] and freely accessible web tools SwissADME [44], pkCSM-pharmacokinetics [45], and SwissTargetPrediction [46]. 

The PubChem web database [43] allowed to obtain for each compound canonical SMILES, a simplified molecular-input line entry specification nomenclature [50] (Table 2), and physicochemical properties such as molecular weight (MW), XLogP3-AA (lipophilicity), rotatable bond count (RBC), topological polar surface area (TPSA), hydrogen bond donor count (HBDC), hydrogen bond acceptor count (HBAC), and complexity (reported in Table 3).

For each compound, the canonical SMILES were entered into the web tools SwissADME [44], which assembles the most relevant computational methods to provide a global appraisal of the pharmacokinetics profile of small molecules [50,51], and pkCSM-pharmacokinetics [45], a novel method for predicting/optimizing small-molecule pharmacokinetic and toxicity properties [52]. 

**Figure 7:**
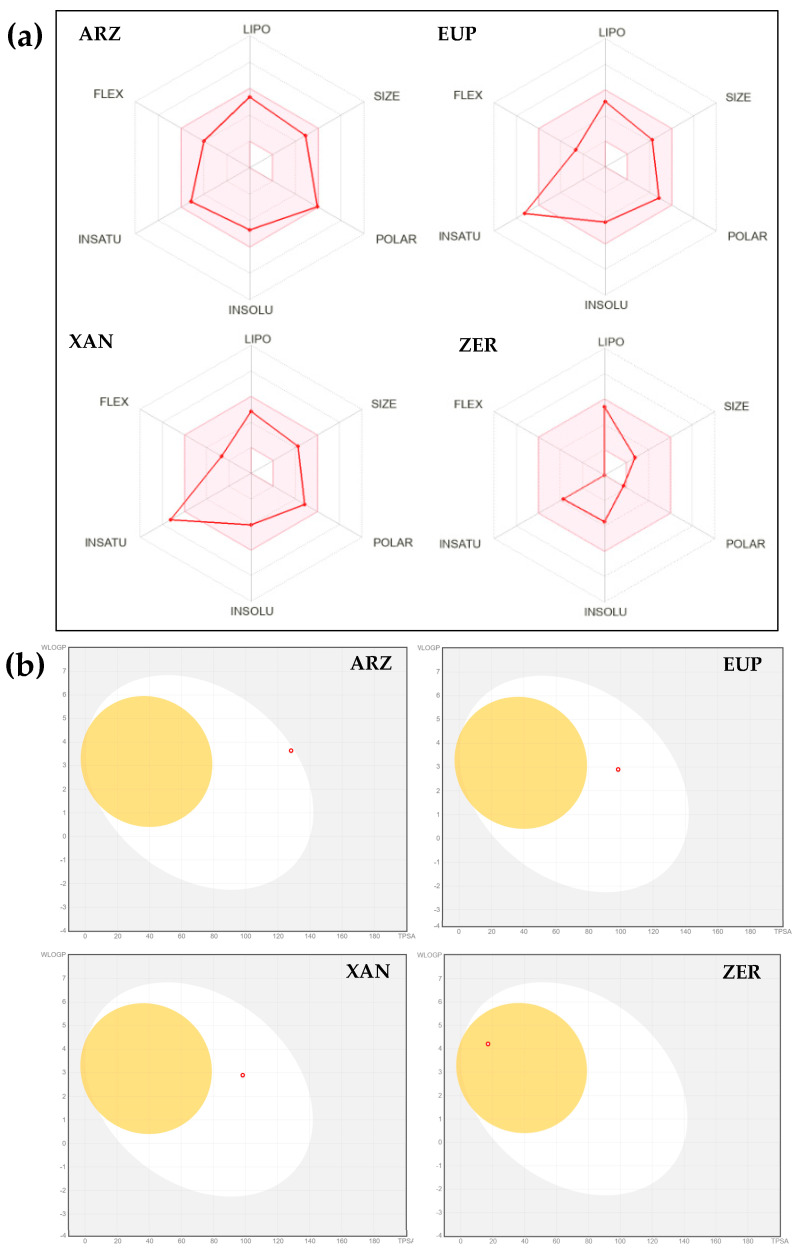
Bioavailability radars (**a**) and “BOILED-Egg” graphs (**b**) by SwissADME web tool [44,51], computed for arzanol (ARZ), eupatilin (EUP), xanthomicrol (XAN), and zerumbone (ZER).

The most relevant physicochemical/pharmacokinetic properties of XAN, ZER, EUP, and ARZ including Consensus Log P_o/w_ (the logarithm of the n-octanol/water partition coefficient, lipophilicity), values of Log S (ESOL), Log S (Ali), Log S (SILICOS-IT) (water solubility), human intestinal absorption (HIA, bioavailability), blood–brain barrier (BBB) permeability, and central nervous system (CNS) permeability) are listed in Table 3. The Bioavailability Radar, a graphical representation of the drug-likeness, and the “BOILED-Egg” representation of passive gastrointestinal absorption and BBB penetration, obtained for tested compounds by SwissADME [44,51], are reported in Figure 7a and Figure 7b, respectively.

The Bioavailability Radar (Figure 7a) by SwissADME allows a first description of the drug-likeness of a molecule and, in this representation, the pink area represents the optimal range for each property, corresponding to Log P_o/w_ (specifically XLogGP3) between −0.7 and + 5.0, (lipophilicity), MW between 150 and 500 g/mol (size), TPSA between 20 and 130 Å^2^ (polarity), log S not higher than 6 (water solubility), fraction of carbons in the sp^3^ hybridization not less than 0.25 (saturation), no more than 9 rotatable bonds (flexibility) [44,51]. 

In the intuitive “BOILED-Egg” representation (Figure 7b) for a molecule (dot) the position in the white region indicates a high probability of passive absorption by the gastrointestinal tract (IA) (bioavailability), and the position in the yellow region (yolk) signifies a high probability of brain penetration and brain access (BBB penetration) [44,51]. Moreover, red dots indicate molecules predicted not to be a substrate of glycoprotein P (P-gp) [44,51]. 

MW value of 402.4, values of 3.9 (XLogP3-AA) and 3.42 (Consensus Log P_o/w_) for lipophilicity, values of Log S (water solubility) from −4.70 to −6.28, 6 rotatable bonds, a total number of hydrogen bonds (THB, donor + acceptor bonds) of 11, a TPSA of 124 Å^2^, and a complexity value of 757 were computed for ARZ, indicating mid-polarity properties of the compound [10,43,44,45]. A high bioavailability was predicted for this phloroglucinol, and a value of 74.92% was estimated for its intestinal absorption (IA). No BBB permeability (BOILED Egg model) (Figure 7b) was predicted for this phenol, while values of −1.204 (log BB) and −2.935 (log PS) were calculated for BBB and CNS permeability, respectively. 

Observing the Bioavailability Radar (Figure 7a), ARZ was predicted as orally bioavailable, entering into the optimal range (pink area).

Value of 344.3 for MW, values of 2.9 (XLogP3-AA) and 2.54 (Consensus Log P_o/w_) for lipophilicity, values of Log S from −4.33 to −5.33 (moderately soluble), four rotatable bonds, nine THB donor + acceptor bonds, TPSA value of 94.4 Å^2^, a complexity value of 520, a high bioavailability (IA = (78.996%), no BBB permeability (BOILED Egg model), and values of −0.809 (log BB) and −3.083 (log PS) of BBB and CNS permeability, respectively, were computed for the methoxyflavone EUP [43,44,45]. The Bioavailability radar qualified this phenolic compound as orally bioavailable, mostly entering into the pink area.

XAN is a chemical analog of EUP and is characterized by the same values for MW, XLogP3-AA, HBDC, HBAC, RBC, TPSA, identical Bioavailability radar, and no BBB permeability in the BOILED Egg model. Values of 2.42 for Consensus Log P_o/w_, from −4.4 to −5.33 for Log S (indicated as moderately soluble), 96.278% for IA, −0.575 (log BB) for BBB permeability, and −3.295 (log PS) for CNS permeability were computed for this methoxyflavone [43,44,45].

Value of 218.33 for MW, values of 3.9 (XLogP3-AA) and 3.57 (Consensus Log P_o/w_) for lipophilicity, values of Log S from −3.68 to −4.0 for water solubility (indicated as soluble), no rotatable bonds, 1 HBAC, TPSA value of 17.1 Å^2^, a complexity value of 354, a high bioavailability (IA = 95.781%), and values of 0.522 (log BB) and −2.647 (log PS) of BBB and CNS permeability, respectively, were predicted for the sesquiterpene ZER [43,44,45]. The Bioavailability radar indicated that ZER was entirely inside the pink area, qualifying it for a good bioavailability profile. Moreover, in the BOILED Egg model, ZER appeared in the yellow region (yolk) with a red point, indicating a good BBB permeability and a high probability of brain penetration. 

**Figure 8:**
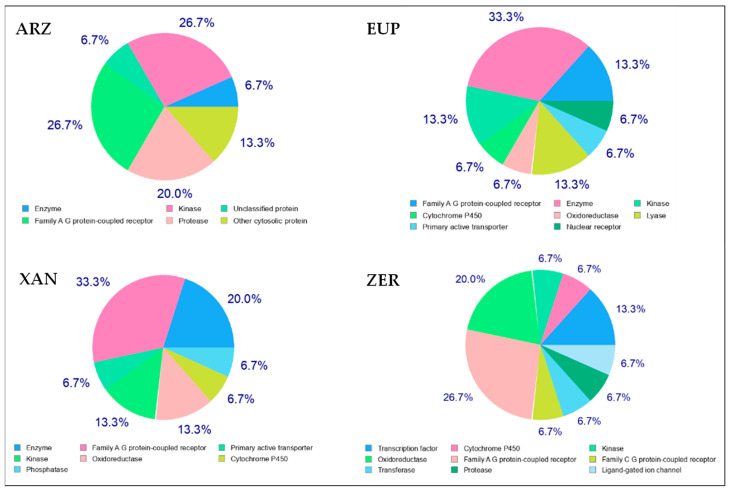
Summary of target classes by the SwissTargetPrediction web tool [46,53] computed for arzanol (ARZ), eupatilin (EUP), xanthomicrol (XAN), and zerumbone (ZER).

The introduction of canonical SMILES of tested compounds into the web tool SwissTargetPrediction [46], a common database for ligand-based target prediction, allowed us to obtain an indication of their most probable protein targets [53]. SwissTargetPrediction is a combined two-dimensional (2D) and 3D ligand-based similarity method that performs target prediction by calculating the similarity between query molecule/ligand sets in a reference database [46,54]. Figure 8 reports the principal predicted target protein classes (among the top 15) for ARZ, EUP, XAN, and ZER.

The results demonstrated that ARZ targeted several molecular/biochemical pathways, including G-protein coupled receptor (26.7%), kinase (26.7%), protease (20.0%), and unspecified cytosolic protein. 

The predicted target classes for EUP were G-protein coupled receptor (13.3%), kinase (13.3%), lyase (13.3%), cytochrome P450, oxidoreductase, primary active transporter, and nuclear receptor. 

The target classes predicted for XAN were similar to those of EUP, including G-protein coupled receptor (33.3%), kinase (13.3%), oxidoreductase (13.3%), cytochrome P450, primary active transporter, and phosphatase. 

ZER showed the most complex profile of probable protein targets among the tested compounds, including G-protein coupled receptor (26.7%), oxidoreductase (20%), transcription factor (13.3%), cytochrome P450, kinase, transferase, protease, and ligand-gated ion channel. 

### 2.5. Correlation Between Computed Properties of ARZ, EUP, XAN, ZER, and Cytotoxicity and Apoptosis Induction 

The cytotoxic and pro-apoptotic effects of the anticancer compounds ARZ, EUP, XAN, and ZER on SH-SY5Y neuroblastoma cells were correlated with their physicochemical and pharmacokinetic properties to identify the drug properties mainly affecting antitumor activity in neuroblastoma cells. 

**Figure 9:**
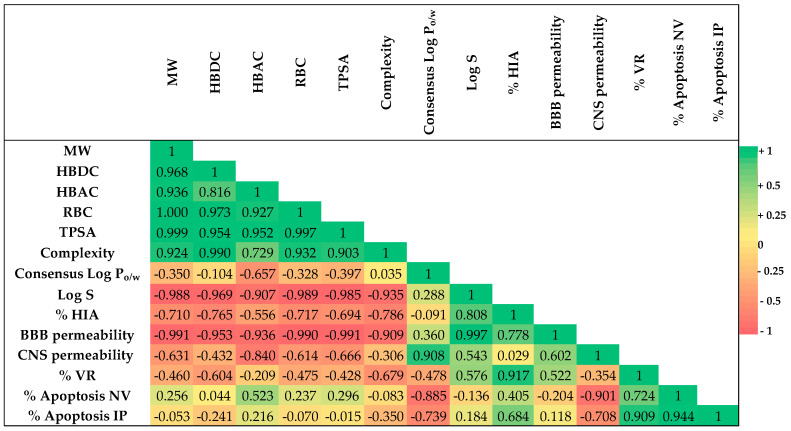
Heatmap of Pearson’s correlations (r) calculated between computed properties of arzanol (ARZ), eupatilin (EUP), xanthomicrol (XAN), and zerumbone (ZER) reported in Table 3 and mean values of cytotoxicity (% VR) and apoptosis induction (% Apoptosis NV and % Apoptosis IP) determined at the dose of 50 μM.

Figure 9 shows the heatmap of Pearson’s correlations (r) calculated between computed properties of ARZ, EUP, XAN, and ZER reported in Table 3, including MW, HBDC, HBAC, RBC, TPSA, complexity, Consensus Log P_o/w_ for lipophilicity, Log S (as the mean value of ESOL, Ali, and SILICOS-IT), HIA, BBB permeability, and CNS permeability, and their cytotoxic (% VR) and apoptosis induction (% Apoptosis NV and % Apoptosis IP) effects obtained at the dose of 50 μM. 

The heatmap analysis color scheme indicates the strength and direction of correlations: red represents a strong negative correlation, light orange represents a weak negative correlation, light green represents a weak positive correlation, and green represents a strong positive correlation. 

Regarding cytotoxic activity (% VR), from the heatmap correlation matrix, a strong positive correlation was determined between % VR/% HIA (r = 0.917), highlighting a potential role of bioavailability in SH-SY5Y cancer cell growth inhibitory effect of the four tested compounds (Figure 9 and Figure S3a). Negative correlations emerged between % VR/complexity (r = −0.679) (Figure 9 and Figure S3b) and % VR/HBDC (r = −0.604). 

Negative correlations were measured between % Apoptosis NV/Consensus Log P_o/w_ (r = −0.885) (Figure 9 and Figure S3c), % Apoptosis IP/Consensus Log P_o/w_ (r = −0.739) (Figure 9 and Figure S3d), evidencing a negative modulation of compound lipophilicity in the cancer cell apoptosis induction. A weak positive correlation was also determined between % Apoptosis IP/% HIA (r = 0.684).

A positive correlation emerged between the cytotoxicity of compounds and their ability to induce apoptosis, and values of r = 0.724 and r = 0.909 were calculated for % VR/% Apoptosis NV and % VR/% Apoptosis IP, respectively. Moreover, a strong correlation (r = 0.944) emerged between the results obtained with the two apoptotic assays.

## 3. Discussion

Plant natural extracts and isolated metabolites (phytochemicals) represent a significant source of new therapeutic drugs for the treatment of different types of cancers [1,2,3,55,56]. However, the development of natural product-based anticancer agents and their application in clinical trials requires overcoming significant problems such as medication resistance, systemic toxicity, limited absorption, and complete knowledge of the detailed mechanisms underlying the anticancer properties [1,2,3,55,56].

Neuroblastoma is a type of cancer of immature nerve cells originating in the adrenal gland, nerve ganglia, or neck, and it is one of the most common pediatric extracranial solid tumors that compromise the health of children [39,40,41]. Currently, multiple therapies have been applied in the clinical treatment of neuroblastoma, including chemotherapy [41]. Overcoming obstacles such as chemoresistance, organ toxicity, and other side effects observed in patients receiving standard chemotherapy (doxorubicin, etoposide, and cyclophosphamide) requires the discovery of novel therapeutic agents [39,40,41].

In this study, the anticancer activity of the natural plant-derived compounds ARZ, EUP, XAN, and ZER was investigated and compared in neuroblastoma SH-SY5Y cells, a cancer cell line amply used as a model to study neuroblastoma-specific therapeutics [42]. 

Moreover, the anticancer profiles of the compounds were correlated with their calculated physicochemical and pharmacokinetic properties to identify the potential drug properties mainly affecting antitumor activity in neuroblastoma cells. 

The anticancer properties of compounds were first assessed by monitoring the effects on SH-SY5Y cancer cell viability (by MTT assay) and morphology after 24 h of incubation at the dose range of 2.5–100 μM. We previously used the same experimental conditions to assess the cytotoxic effect of ARZ [5,10,11,12,13], EUP [17,20,27], XAN [20,27], and ZER [35] in other normal and cancer cells. EUP, XAN, and ZER induced a dose-dependent decrease in cancer SH-SY5Y cell viability compared to control cells, and the order of potency was XAN > ZER > EUP. ARZ did not inhibit neuroblastoma SH-SY5Y cell growth after 24 h of incubation, as previously observed [12]. 

In our experimental conditions, the methoxyflavone XAN emerged as the most cytotoxic compound (IC_50_ = 22.8 μM). The values of % viability reduction and the order of potency obtained for XAN, ZER, EUP, and ARZ in SH-SY5Y cancer cells at 50 μM were comparable with those previously obtained for the four compounds in human cervical cancer HeLa cells in similar experimental conditions (24 h of incubation, MTT assay), with XAN showing the highest cytotoxicity (45% viability reduction), followed by ZER (34%), EUP (32%), and ARZ (4%) [13,17,27,35]. 

According to our results, previous studies evidenced the superior cytotoxicity (24 h of incubation) of XAN in comparison to the chemical analog EUP in cervical cancer cells HeLa (IC_50_ values of 182 μM and > 200 μM for XAN and EUP, respectively) [27] and in A375 melanoma cells in the range 2.5–10 μM [20]. An IC_50_ value of 35 μg/mL (101.6 μM) was previously reported for XAN after 24 h incubation in 4T1 cancer cells [29], whereas IC_50_ values ranging from 4.5 to 40.6 μg/mL (13–124 μM) were determined in AGS, WEHI-164, HL60, SaOs-2, and HT29 cancer cells after 72 h of incubation (Table 1) [25]. 

The cytotoxicity values obtained for EUP in our experimental conditions were similar to those obtained in A375 melanoma cells (at 24 h incubation) [20] and Ect1/E6E7 (48 h) [15], but lower than those previously observed after 48 h incubation in cancer HeLa, HCT116, HT29, Hec1A, and KLE, cells [15,18,48].

We previously demonstrated a less marked cytotoxic effect of XAN and EUP in 3T3 fibroblasts with respect to cancer HeLa cells, indicating more selective toxicity towards malignant cells than normal cells [27]. A higher toxicity was observed for XAN and EUP in A375 skin melanoma cells than in normal HaCaT keratinocytes [20]. Moreover, certain cytotoxicity was previously observed for XAN in normal human fetal foreskin fibroblasts HFFF-P16 (162.4 μM after 72 h of incubation) [25].

Our data on ZER cytotoxicity in cancer SH-SY5Y cells (IC_50_ = 60 μM) are in line with previous results obtained in Caco-2 (24 h incubation), SW480 (48 h), MCF-7 (72 h), and MDA-MB-231 (72 h) cells, nevertheless lower than those reported in B16F10 (24 h) and HeLa 4 (48 h) cancer cells (Table 1) [35,37,49]. ZER demonstrated antiproliferative activity towards several cancer cell lines but only marginal effects on normal cells [31,33,35].

Variable cytotoxic activity was reported for ARZ in cancer cells at 24 h incubation, strictly depending on the type of cells [8,13,14,35]. The phloroglucinol showed the ability to decrease viability in various cancer cell lines (A549, RT-112, HeLa, undifferentiated CaCo-2) and B16F10 melanoma cells [8,13,14], without cytotoxic effects in peripheral blood mononuclear cells [8], immortalized Vero cell line [10], and differentiated CaCo-2 cells (a model of the intestinal epithelium) [10], indicating its selective cytotoxicity vs. tumoral cells. 

According to the observed cytotoxic effect, contrast phase microscopic observation of SH-SY5Y cancer cells incubated for both 2 h and 24 h with XAN, ZER, and EUP evidenced marked changes in the cell morphology such as a reduced cell density, an increase in the number of rounded and granulated cells, and membrane blebbing, highlighting evident signs of an apoptotic process. Some rounded cells were observed in neuroblastoma cells treated for 2 h with ARZ, whereas low effects on cell morphology were evidenced after 24 h incubation, coupled with no effect on cell viability, probably due to a possible cell recovery of ARZ-induced toxic damage at long incubation times with the compound.

The short-time treatment (2 h pre-incubation) of neuroblastoma SH-SY5Y cells with all compounds determined a significant dose-dependent increase in the number of apoptotic cells vs. control cells, evaluated by NV assay (green fluorescence), able to detect caspase-3/7 activity inside cells [12,20], and PI fluorescence assay (red fluorescence), which evidences late apoptotic and necrotic cells [12]. 

XAN exhibited the highest activity in both models, with an evident induction of apoptosis at the dose of 25 μM. The apoptotic effect of XAN was evidenced in various cancer cell lines [20,23,26,27,29,30]. According to our results, the 24 h-treatment with XAN has been previously demonstrated to induce in A375 melanoma cells a significant marked increase, vs. control cells, in the number of NV-stained cells at the doses of 10 μM (482% of green fluorescence intensity) and 25 μM (566%) [20]. XAN, at 15 and 21 μM, greatly increased the percentage of both early and late apoptotic cells (by annexin/PI test) in human colon cancer HCT116 cells [30]. Moreover, XAN induced a concentration-dependent apoptotic (staining with acridine orange) and necrotic cell death (PI assay) in the HL60 cell line after 4 h of incubation at the dose range 20–250 ng/mL [26].

An evident induction of apoptosis was observed from 25 μM also for EUP in neuroblastoma SH-SY5Y cells, whereas this methoxyflavone was less active than the chemical analog XAN. In previous research conducted in cancer HeLa cells, we demonstrated, by flow cytometry, immunofluorescence, and fluorescence microscopy, the EUP capacity (at the doses 25 and 50 μM) to induce apoptosis (presence of rounded cells, apoptotic bodies, and membrane blebbing) after 24 h of incubation [17]. Moreover, we recently assessed the occurrence of apoptosis (by NucView 488 assay) in A375 melanoma cells at 25 μM (value of 339% of controls) after 24 h incubation, also exhibiting in this model a lower potency than XAN [20]. EUP treatment (40 µM) significantly increased the percentage of Hoechst-stained positive cells (apoptosis rate) in different cervical cancer cell lines (HeLa and Caski) after 48 h incubation [15]. EUP significantly increased apoptosis at 50 μM in HCT116 cells (4.4-fold) and in HT29 cells (1.6-fold) [18]. 

ZER induced a dose-dependent increase in green (NV assay) and red (PI assay) fluorescence emission in neuroblastoma SH-SY5Y cells after 24 h of incubation at the concentration range 25–100 μM. Induction of apoptosis has been indicated as the primary mechanism underlying the anti-proliferative activity of ZER, as observed in many tumor cell lines [32,33,34,35,37,38]. We previously evidenced a series of morphological changes/features of apoptosis in the ZER-treated HeLa cells (50 and 100 μM ZER for 24 h), including loss of cell volume or cell shrinkage, plasma membrane blebbing, condensed nuclei, loss of mitochondrial membrane potential, mitochondrial fragmentation, and swelling [35]. A significant dose-dependent increase in the apoptotic cell population was observed in SW480 colorectal cancer cells treated for 48 h with ZER (50, 75, and 100 μM) by flow cytometry analysis using annexin V-fluorescein isothiocyanate/PI staining [37].

Despite no cytotoxicity and low effects on cell morphology after 24 h incubation, ARZ induced at 2 h incubation a significant increase in the green fluorescence from 50 μM (NucView 488 assay) and red fluorescence (PI assay) at 100 μM in SH-SY5Y cells, indicating cell apoptosis and necrosis. To the best of our knowledge, no previous study reported the apoptotic effect of ARZ in cancer cells. However, ARZ has been indicated as a lead compound for drug development in cancer therapy for its ability to modulate autophagy, a lysosomal pathway that plays a role in different human pathologies, including cancer [14]. A strong interaction exists between autophagy and apoptosis, and autophagy can trigger cell death by promoting apoptosis [57].

The values of cytotoxicity (% VR) and apoptosis induction (% Apoptosis NV and % Apoptosis PI) obtained for the four compounds in neuroblastoma SH-SY5Y at 50 μM were correlated to their physicochemical and pharmacokinetic properties to identify those mainly affecting the observed activities in neuroblastoma cells.

From the heatmap correlation matrix, the compound effects on cell growth/viability emerged as strongly positively correlated to the percentages of the tested compounds predicted to be absorbed through the human intestine (% HIA) using pkCSM-pharmacokinetics (% VR/% HIA, r = 0.917) [45,50,52]. The observed relation highlighted the role of the compound bioavailability/capability to cross biological membranes in the SH-SY5Y cancer cell cytotoxicity of the four tested compounds. 

In a cell model, the bioavailability of a small molecule depends on several determinants, including physicochemical properties, formulation, and biological factors [58]. Among physicochemical properties, solubility determines the dissolution rate and maximum absorbable dose, lipophilicity (log P) affects membrane permeability and distribution, molecular size/weight influence passive diffusion and active transport, and pKa affects ionization state/absorption [58]. Among biological factors, efflux transporters affect cellular uptake and retention [58].

Lipophilicity is one of the key properties of a potential drug that crucially determines the solubility, the ability to penetrate biological membranes, and transport to the molecular target [58,59]. A general guide for good oral bioavailability (good permeability and solubility) is to have a moderate Log P (0 < Log P < 3) [50]. Recently, a Log P between 1 and 3 has been indicated as favorable for oral bioavailability, balancing membrane permeability with aqueous solubility [58]. Anticancer lipophilic compounds often rely on entering cells via lipid membranes and directly affect intracellular structures and organelles (cytoskeleton, mitochondria), modulating intracellular signaling pathways and molecular targets.

The methoxylated flavone XAN, the most bioavailable compound by pkCSM-pharmacokinetics (HIA = 96.278%), emerged as the most cytotoxic on neuroblastoma SH-SY5Y cells, while the non-toxic compound ARZ was characterized by the lowest value of bioavailability (HIA = 74.92%). XAN is a trimethoxylated hydroxyflavone characterized by a MW value of 344.3, a certain hydrophobicity (values of 2.9 and 2.42 for XLogP3-AA and Consensus Log P_o/w_, respectively), complexity value of 512, values from −4.4 to −5.33 for Log S (moderately soluble), TPSA value of 94 Å^2^, four rotatable bonds (flexibility), 9 THB donor + acceptor bonds, mostly entering into the Bioavailability radar pink area [43,44,45]. 

These physicochemical characteristics crucially influence XAN interactions with SH-SY5Y cell membranes [20,27,60]. For flavonoids, the Log P value has been correlated with the extent of their interaction with biological membranes [27,61]. It has been proven that flavonoids possess the ability to localize either in the hydrophobic core of the membrane lipid bilayer or at the cell lipid membrane surface in relation to their chemical properties and hydrophobicity [61]. Hydrophobic flavonoids partition deeper into the lipid bilayer, thereby disrupting the compact packing of lipids [61]. The membrane interactions and localization of flavonoids are fundamental in altering membrane-mediated cell signaling pathways [61]. 

The uncommon plant lipophilic methoxylated flavones have been demonstrated to possess chemopreventive properties superior to unmethylated flavonoids or polyphenols [23,24,25,60]. The methylation of free phenolic groups (-OH) on the flavone skeleton has been demonstrated to improve the ability of derivatives to cross cell membranes, oral bioavailability, and metabolic stability [60]. The cytotoxic activity of the methoxylated flavone XAN in cancer cells has been related to its ability to induce cell cycle arrest [23,27,29,30], prooxidant effects [20], lipid profile modulation [20,27], and apoptosis through the modulation of a variety of molecular targets and signaling pathways [20,23,26,27,29,30]. 

The apoptotic activity in neuroblastoma in SH-SY5Y cells emerged as negatively correlated with the lipophilicity of the compounds, as indicated by the correlation coefficients determined between % Apoptosis NV/Consensus Log P_o/w_ (r = −0.885) and % Apoptosis IP/Consensus Log P_o/w_ (r = −0.739). XAN, the most apoptotic compound in both systems, was characterized by the lowest value of lipophilicity among the tested compounds. Values of Log P reported for XAN fall within the indicated optimal range, balancing membrane permeability with aqueous solubility [58]. The XAN physicochemical characteristics conferred to this compound the capacity to interact with the neuroblastoma cell membrane, inducing apoptosis, maybe through interactions with proteins/signaling pathways in the aqueous compartments inside the cells (intrinsic pathways) [62]. A previous study evidenced the XAN anti-proliferative effect in breast cancer due to its up-regulation of the Bcl2 family and Bax protein [23]. Moreover, the increase in caspase-9 expression was demonstrated after XAN treatment in a breast cancer mouse model, highlighting the potential role of XAN in the activation of the mitochondrial pathway of apoptosis [29]. 

XAN emerged as the most active compound in cancer SH-SY5Y cells. In addition to good membrane permeability, XAN was predicted by the Swiss-ADME not to be a substrate of glycoprotein P (P-gp), an ATP-dependent drug-extracting pump found in various human tissues [44,50,51]. Efflux transporters, such as P-gp, play a role in controlling the transport of chemotherapeutic agents in some cancers, including neuroblastoma, limiting their bioavailability and distribution across the BBB and contributing to chemoresistance [39,41,51,58]. Therefore, a drug that does not bind to P-gp may have several advantages, including increased bioavailability and therapeutic efficacy, and reduced side effects by minimizing drug release in non-target tissues [58].

Our results demonstrated the anticancer properties of XAN in SH-SY5Y cancer cells through cell growth inhibition and apoptotic mechanisms, highlighting its potential application in the treatment of neuroblastoma, an extracranial tumor of the peripheral nervous system [39].

The study of bioactive molecule permeability across the BBB is essential to screening the capacity of neuroprotective compounds to reach the CNS [63,64]. Molecular parameters, such as MW, TPSA, and lipophilicity, have been reported to affect diffusion across this physiological barrier [63,64]. Due to the lipophilic nature of the BBB, molecules with higher Log P values have been proposed to penetrate better through the BBB [63,64]. For CNS drugs, a high Log P range (2–5) may be optimal to cross the BBB [12,50,64]. In the pkCSM-pharmacokinetics model, molecules with a log BB < −1 are considered poorly distributed to the brain, while compounds with values > 0.3 readily cross the BBB [12,45,52]. Moreover, compounds with a log PS > −2 are considered to penetrate the CNS, while those with log PS < −3 are considered unable to penetrate the CNS [12,45,52].

No BBB permeability (by passive diffusion) was predicted for XAN by the Swiss-ADME (BOILED Egg model) [44,51]. Values of −0.575 (log BB) and −3.295 (log PS) were calculated for BBB and CNS permeability, respectively, by pkCSM-pharmacokinetics for XAN [45], indicating a low computed BBB and CNS permeability. XAN was predicted to passively not cross the BBB; however, a carrier- or receptor-mediated transport through the BBB cannot be excluded. 

Despite lower activity than XAN, EUP showed interesting anticancer properties on SH-SY5Y cells. This methoxyflavone shares with XAN various physicochemical properties; however, differences observed in the bioactivity and computed pharmacokinetic/pharmacological properties could be ascribable to the different substitution patterns in the C6-C3-C6 rings, which affect the interactions with cell membranes and molecular targets [60,61].

Also, ZER showed a significant cytotoxicity and apoptotic effect in neuroblastoma SH-SY5Y cells. This sesquiterpene exhibited lower values of MW, TPSA, and complexity than other tested compounds, and high hydrophobicity (values of 3.9 and 3.57 for XLogP3-AA and Consensus Log Po/w, respectively), perfectly entering into the Bioavailability radar pink area [43,44]. An excellent BBB permeability (by passive diffusion) was predicted for ZER by the Swiss-ADME (BOILED Egg model) [44,51], and values of BBB permeability of 0.522 (log BB) and CNS permeability of −2.647 (log PS) were computed by pkCSM-pharmacokinetics for this natural compound [45], qualifying it as potential CNS tumor therapeutic for in vivo application.

This study demonstrated the ability of XAN, ZER, and EUP to reduce viability and induce apoptosis in SH-SY5Y cells, highlighting their potential role as a therapeutic strategy for the treatment of neuroblastoma, a tumor that grows outside the CNS. 

In the search for new anticancer drugs, the criteria of cytotoxicity activity established by the United States National Cancer Institute (NCI) indicate that only those extracts that present IC_50_ values below 30 µg/mL and pure compounds with IC_50_ values below 4 µg/mL in tumor cell line assays are considered to be promising for anticancer drug development [65]. The IC_50_ value of XAN (7.9 μg/mL) is near the indicated value, qualifying this compound as a promising cytotoxic agent in neuroblastoma. 

Limitations of this study include the use of a single cell line, the absence of exploration of detailed molecular mechanisms of compounds in SH-SY5Y cells, and the lack of evaluation of their synergic effects and in vivo effects, which are necessary to confirm the efficacy and safety of the compounds. 

## 4. Materials and Methods

### 4.1. Materials

Chemicals for cell culture, including penicillin, streptomycin, fetal bovine serum (FBS), Dulbecco’s Modified Eagle’s Medium (DMEM), and Trypsin 0.25%-EDTA, were acquired from EuroClone (Pero, MI, Italy). Dimethyl sulfoxide (DMSO) and 3-(4,5-dimethylthiazol-2-yl)-2,5-diphenyltetrazolium bromide (MTT) were purchased from Merck Life Science (Milan, Italy). NucView^®^ 488 (NV) was purchased from Biotium (Fremont, CA, USA). Gemcitabine hydrochloride (2′-Deoxy-2′,2′-difluorocytidine) was bought from Sigma-Aldrich (Milan, Italy). Propidium iodide (PI) was acquired from Thermo Fisher Scientific (Waltham, MA, USA). 

### 4.2. ARZ, EUP, XAN, and ZER Extraction and Isolation

ARZ was extracted and isolated (final purity was 98%) from the dried leaves and flowerheads of *H. italicum* ssp. *microphyllum* as previously reported [12]. According to the literature, EUP (98% purity) and XAN (98% purity) were isolated from the Swiss chemotype of *A. umbelliformis* Lam. (Asteraceae) [17,20] and *A. erba-rotta* subsp. moschata (Wulfen) I. Richardson (musk yarrow, flowering tops) [20,27], respectively. ZER was isolated from the essential oil of shampoo ginger by direct crystallization, as previously reported [35]. All compounds were characterized by the ^1^H NMR spectroscopic method, and the structures were confirmed by comparing the data with related scientific literature [4,5,12,17,20,27]. ^1^H NMR spectra of XAN, ZER, EUP, and ARZ are reported in Figures S4, S5, S6 and S7, respectively. The solutions of compounds were freshly prepared before all experiments, and all compounds remained stable under experimental conditions, as previously reported [8,34,66,67]. 

### 4.3. Physicochemical/Pharmacokinetic Properties and Protein Targets Prediction of ARZ, EUP, XAN, and ZER by In Silico Evaluation 

Physicochemical properties (MW, XLogP3-AA, HBDC, HBAC, RBC, TPSA, and complexity) and simplified molecular-input line entry specification (canonical SMILES) nomenclature of ARZ, EUP, XAN, and ZER were obtained from the free web database PubChem [43]. The canonical SMILES of the compounds were introduced into the freely accessible web tools SwissADME [44], pkCSM-pharmacokinetics [45], and SwissTargetPrediction [46]. The SwissADME web tool, which provides a global evaluation of the pharmacokinetics profile of small molecules, was used to estimate compound lipophilicity (Consensus Log P_o/w_), water solubility (Log S), gastrointestinal absorption (according to the white of BOILED-Egg model), and BBB permeation (according to the yolk of BOILED-Egg model) [44,50,51]. The pkCSM-pharmacokinetics, a method for predicting and optimizing small-molecule pharmacokinetics/toxicity properties, was employed to calculate compound intestinal absorption (IA, %), the logarithm of the ratio of compound concentration in the brain and the blood (log BB), and the logarithm of blood-brain permeability-surface area product (log PS) [45,50,52]. The SwissTargetPrediction web tool was used for the prediction of the most probable protein targets of ARZ, EUP, XAN, and ZER (the analysis was restricted to the top 15 Homo sapiens targets) [46,53].

### 4.4. SH-SY5Y Neuroblastoma Cell Culture

Human neuroblastoma cell line SH-SY5Y was supplied by the Cell Bank Interlab Cell Line Collection, IRCCS San Martino Policlinico Hospital, Genova, Italy (code# HTL95013). Neuroblastoma cells were grown in a DMEM high glucose medium enriched with 10% *v*/*v* FBS and 100 units/mL of streptomycin/penicillin, in a humidified atmosphere of 5% CO_2_ at 37 °C. The cells were propagated by changing the medium every two days and split weekly when confluent using a Trypsin 0.25%-EDTA solution. 

### 4.5. Cell Viability (MTT Assay)

The cytotoxic effect of ARZ, EUP, XAN, and ZER was measured in cancer SH-SY5Y cells by the MTT viability colorimetric assay, as previously reported [11,12,17,20]. The effect of different amounts of DMSO (vehicle used to dissolve the compounds) on cell viability was also evaluated. SH-SY5Y neuroblastoma cells were seeded (at a density of 10^5^ cells/mL) in 96-well plates in a complete culture medium (100 μL). After 48 h incubation, SH-SY5Y cells were treated with ARZ, EUP, XAN, ZER (2.5–100 μM, from a 10 mM solution in DMSO) or DMSO (from 0.025 to 2% *v*/*v*) in a complete fresh medium for 2 h and 24 h, whereas control cells (untreated-cells) were cultured for 2 and 24 h in a complete fresh medium. The cells were also treated for 2 and 24 h with the anticancer compound gemcitabine [47] (2.5–100 μM, from a 10 mM water solution) as a positive control to assess test validity. Then, after medium removal, control (non-treated) cells, compound-treated cells, and cells incubated with DMSO (vehicle-treated cells) were subjected to the MTT assay as previously reported [11,12]. The auto microplate reader (Infinite 200, Tecan, Austria) was used to measure color development at the wavelength of 570 nm. Results of cell absorbance, proportional to the number of viable cells, were expressed as a percentage of cell viability compared to control (untreated) cells (100% viability). 

Morphological observations of control cancer SH-SY5Y cells and cells after 2 and 24 h of incubation with various amounts of compounds and DMSO were performed by microscopic analysis with a ZOE™ Fluorescent Cell Imager (Bio-RadLaboratories, Inc., Hercules, CA, USA). 

### 4.6. NucView and Propidium Iodide Apoptosis Assays

The effect of ARZ, EUP, XAN, and ZER on cell apoptosis and cell death/necrosis was assessed by staining cancer SH-SY5Y cells with NucView 488 (NV) and propidium iodide (PI) dyes, as previously reported [11,12,20]. NV, a substrate of the enzyme caspase-3, detects the activity of caspase-3/7 within cells and profiles apoptotic cells with green fluorescence [11]. PI is a DNA-binding fluorescent dye (red) that enters cells with disrupted membranes and can evidence necrotic and late apoptotic cells [12].

SH-SY5Y neuroblastoma cells were seeded in 96-well plates (at a density of 10^5^ cells/mL) in 100 μL/well of complete culture medium. After 48 h incubation, SH-SY5Y cells were incubated with ARZ, EUP, XAN, ZER (25, 50, and 100 μM, from a 10 mM solution in DMSO), GEM (positive control) (25, 50, and 100 μM, from a 10 mM solution in water), and DMSO (1% *v*/*v*, the maximal non-toxic dose used to dissolve compounds) in a complete fresh medium for 2 h. Control (non-treated) cells were pre-incubated for 2 h in a fresh medium. After incubation, SH-SY5Y cells were incubated with PI (final concentration 1 μg/mL) and, in a separate set of samples, with NV, according to the guidelines provided by the manufacturer. After dark incubation with PI (2 h) and NV (3 h), microscopic observations were made using a ZOE™ Fluorescent Cell Imager. The offset values and instrument gain were adjusted using control cells. ImageJ software (version 1.53e) was used for the evaluation of PI and NV images (six different images were processed for each sample). Background fluorescence was subtracted from images, and the fluorescence intensity of treated cells was expressed as % of the control cell fluorescence. 

### 4.7. Pearson Correlation Analysis Between Computed Properties of ARZ, EUP, XAN, and ZER and Their Cytotoxic and Pro-Apoptotic Effects 

Pearson correlation analysis was performed between several computed properties of ARZ, EUP, XAN, and ZER reported in Table 3 including MW, HBDC, HBAC, RBC, TPSA, complexity, Consensus Log P_o/w_, Log S (as mean of ESOL, Ali, and SILICO-IT values), HIA, BBB permeation, and CNS permeation, and cytotoxic (%VR) and pro-apoptotic effects (% Apoptosis NV and % Apoptosis IP) of compounds determined at 50 μM concentration. The correlation analysis statistical method was used to evaluate the strength and direction of the linear relationship between two quantitative variables, resulting in a correlation coefficient that ranged from −1 to +1 [68]. 

### 4.8. Statistical Analyses 

Results were expressed as mean and standard deviation (SD) of three independent experiments involving multiple analyses for each sample. GraphPad Prism version 10.0.0 for Windows (GraphPad Software, Boston, MA, USA) was used to estimate the statistical differences between different data groups. Multiple comparisons of the group means were evaluated by One-way analysis of variance (One-way ANOVA) followed by the Bonferroni Multiple Comparisons Test. The minimal level of significance was *p* < 0.05. Bivariate correlations using Pearson’s coefficient (r) were also performed to evaluate potential correlations between compound cytotoxic and pro-apoptotic effects and computed properties reported in Table 3. 

## 5. Conclusions

The development of new therapeutic approaches for neuroblastoma is strongly mandatory. Our data demonstrated that the incubation of the human neuroblastoma SH-SY5Y cell line for 24 h with XAN, ZER, and EUP greatly affected cell growth and morphology (signs of apoptosis and cell number reduction). At a short time of treatment (2 h of pre-incubation), all compounds induced in SH-SY5Y cells changes in cell morphology and manifested apoptosis and necrosis. In conclusion, the present study highlighted the role of XAN, ZER, and EUP as potential therapeutic strategies or therapeutic coadjutants (ARZ) in neuroblastoma treatment. Moreover, our results furnished information about structural and physicochemical/pharmacokinetic properties involved in compound activity, useful for the development of novel neuroblastoma therapeutic agents. 

Further studies are needed to explore detailed molecular mechanisms of compounds in SH-SY5Y cells, evaluate their synergic effects, and confirm their anticancer effect in vivo neuroblastoma models. 

## Figures and Tables

**Table 1:** Values of IC_50_ (the concentration that decreases the cell viability to 50%) and % viability reduction at 50 μM were determined for arzanol (ARZ), eupatilin (EUP), zerumbone (ZER), and xanthomicrol (XAN) in SH-SY5Y cells and other cancer and normal cells from literature data.

Compound	Cell Line	Type	Method	Incubation Time (h)	% Viability Reduction (at 50 μM)	IC_50_ (μM)	Reference
ARZ	SH-SY5Y	Cancer cells	MTT	24	0	>100	-
	A549	Cancer cells	Trypan blue	24	80	-	[8]
	HeLa	Cancer cells	MTT	24	4	>200	[13]
	B16F10	Cancer cells	MTT	24	11	163	[13,35]
	Caco-2	Cancer cells	AlamarBlue	24	35	88	[13]
	RT-112	Cancer cells	MTT	24	100	6.6/13.2	[14]
	PBMC	Normal cells	MTT	24	0	-	[8]
	HaCaT	Normal cells	MTT	24	0	>100	[11]
EUP	SH-SY5Y	Cancer cells	MTT	24	17	>100	-
	HeLa	Cancer cells	CCK-8 kit	48	≈60 (at 40 μM) ^a^	-	[15]
	Ect1/E6E7	Cancer cells	CCK-8 kit	48	≈25 (at 40 μM) ^a^	-	[15]
	HeLa	Cancer cells	MTT	24	32	>200	[17]
	HCT116	Cancer cells	MTT	48	≈55 ^a^	<50 ^b^	[18]
	HT29	Cancer cells	MTT	48	≈45 ^a^	>50 ^b^	[18]
	A375	Cancer cells	MTT	24	31	>200	[20]
	Hec1A	Cancer cells	MTT	48	≈40 ^a^	82.2	[48]
	KLE	Cancer cells	MTT	48	≈45 ^a^	85.5	[48]
	3T3	Normal cells	MTT	24	18	>200	[27]
	HaCaT	Normal cells	MTT	24	35	>200	[20]
	HESC	Normal cells	MTT	48	≈48 ^a^	65.5	[48]
ZER	SH-SY5Y	Cancer cells	MTT	24	31	69	-
	HeLa	Cancer cells	MTT	24	34	>100	[35]
	B16F10	Cancer cells	MTT	24	50	10	[35]
	Caco-2	Cancer cells	MTT	24	44	73	[35]
	SW480	Cancer cells	MTT	24	≈25 ^a^	160	[37]
	SW480	Cancer cells	MTT	48	≈30 ^a^	102	[37]
	HeLa	Cancer cells	MTT	72	-	18.6	[49]
	MCF-7	Cancer cells	MTT	72	-	66.8	[49]
	MDA-MB-231	Cancer cells	MTT	72	-	70.6	[49]
	Caco-2 ^b^	Normal cells	AlamarBlue	24	20	>100	[35]
XAN	SH-SY5Y	Cancer cells	MTT	24	50	22.8	-
	A375	Cancer cells	MTT	24	27	>200	[20]
	AGS	Cancer cells	MTT	72	-	13.1	[25]
	HT29	Cancer cells	MTT	72	-	123.7	[25]
	HL60	Cancer cells	MTT	72	-	111.8	[25]
	SaOs-2	Cancer cells	MTT	72	-	117.9	[25]
	WEHI-164	Cancer cells	MTT	72	-	95.3	[25]
	HeLa	Cancer cells	MTT	24	45	182.0	[27]
	JIMT-1	Cancer cells	MTT	72	-	99.6	[28]
	4T1	Cancer cells	MTT	24	-	101.6	[29]
	3T3	Normal cells	MTT	24	22	>200	[27]
	HaCaT	Normal cells	MTT	24	37	>200	[20]
	HFFF-P16	Normal cells	MTT	72	-	162.4	[25]

^a^ Deduced from literature figures. ^b^ Monolayers of differentiated Caco-2 cells as a model of intestinal epithelium [35].

**Table 2:** Canonical smiles of arzanol (ARZ), eupatilin (EUP), xanthomicrol (XAN), and zerumbone (ZER) obtained from the PubChem web database [43].

Compound Name	^1^ Canonical SMILES
ARZ	CCC1=C(C(=C(C(=O)O1)CC2=C(C(=C(C(=C2O)CC=C(C)C)O)C(=O)C)O)O)C
EUP	COC1=C(C=C(C=C1)C2=CC(=O)C3=C(O2)C=C(C(=C3O)OC)O)OC
XAN	COC1=C(C(=C2C(=C1O)C(=O)C=C(O2)C3=CC=C(C=C3)O)OC)OC
ZER	C/C/1=C\CC(/C=C/C(=O)/C(=C/CC1)/C)(C)C

^1^ Computed by OEChem 2.3.0 (PubChem release 12 December 2024).

**Table 3:** Physicochemical and pharmacokinetic properties of arzanol (ARZ), eupatilin (EUP), xanthomicrol (XAN), and zerumbone (ZER), computed from the chemical structure and the canonical smiles, obtained from the PubChem database [43] and calculated with the web tools SwissADME [44] and pkCSM-pharmacokinetics [45].

Computed Property	Database/Web Tool	ARZ	EUP	XAN	ZER
Molecular Weight (MW)	PubChem	402.4 g/mol	344.3 g/mol	344.3 g/mol	218.33 g/mol
XLogP3-AA—Lipophilicity	PubChem	3.9	2.9	2.9	3.9
Hydrogen Bond Donor Count (HBDC)	PubChem	4	2	2	0
Hydrogen Bond Acceptor Count (HBAC)	PubChem	7	7	7	1
Rotatable Bond Count (RBC)	PubChem	6	4	4	0
Topological Polar Surface Area (TPSA)	PubChem	124 Å^2^	94.4 Å^2^	94.4 Å^2^	17.1 Å^2^
Complexity	PubChem	757	520	512	354
Consensus Log P_o/w—_Lipophilicity	SwissADME	3.42	2.54	2.42	3.57
Log S (ESOL)—Water Solubility	SwissADME	−4.70	−4.33	−4.04	−3.68
Log S (Ali)—Water Solubility	SwissADME	−6.28	−5.14	−4.67	−4.00
Log S (SILICOS-IT)—Water Solubility	SwissADME	−5.26	−5.33	−5.33	−3.41
**^1^** Gastrointestinal absorption	SwissADME	High	High	High	High
**^2^** Blood–brain barrier (BBB) permeant	SwissADME	No	No	No	Yes
Human Intestinal Absorption (HIA)	pkCSM-pharmacokinetics	74.92%	78.996%	96.278%	95.781%
BBB permeability—Distribution	pkCSM-pharmacokinetics	−1.204 log BB	−0.809 log BB	−0.575 log BB	0.522 log BB
CNS permeability—Distribution	pkCSM-pharmacokinetics	−2.935 log PS	−3.083 log PS	−3.295 log PS	−2.647 log PS

**^1^** BOILED-Egg (white); **^2^** BOILED-Egg (yolk).

## Data Availability

The data that support the findings of this study are available from the corresponding author upon reasonable request.

## Supplementary Materials

The following supporting information can be downloaded at: https://www.mdpi.com/article/10.3390/molecules30081742/s1, Figure S1: Representative phase contrast images of SH-SY5Y cells after 24 h of incubation with the positive control gemcitabine; Figure S2: Representative phase contrast images of SH-SY5Y cells after short-time incubation (2 h) with ARZ, EUP, ZER, and XAN and the positive control gemcitabine; Figure S3: Linear relationships between ARZ, EUP, ZER, and XAN cytotoxic and apoptotic action (at 50 μM) and their computed properties reported in Table 3; Figure S4: ^1^H NMR of XAN in CDCl_3_; Figure S5: ^1^H NMR of ZER in CDCl_3_; Figure S6: ^1^H NMR of EUP in CDCl_3_; Figure S7: ^1^H NMR of ARZ in CDCl_3_.
