# Supplementary material for: Comparative Evaluation of Cytotoxic and Apoptotic Effects of Natural Compounds in SH-SY5Y Neuroblastoma Cells in Relation to Their Physicochemical Properties
Source: Molecules. 2025 Apr 13;30(8):1742. doi: 10.3390/molecules30081742 (PMC12029500; doi:10.3390/molecules30081742)
Supplement: Supplementary file 1 [file molecules-30-01742-s001.zip › molecules-3511416-supplementary.pdf]

molecules

Article

Comparative evaluation of cytotoxic and apoptotic effects of natural compounds in SH-SY5Y neuroblastoma cells in relation to their physicochemical properties

Antonella Rosa [1,*], Federica Pollastro [2], Valeria Sogos [1], and Franca Piras [1]

[1] Department of Biomedical Sciences, University of Cagliari, 09042 Monserrato, Italy; anrosa@unica.it (A.R.); sogos@unica.it (V.S.); fpiras@unica.it (F.P.);
[2] Department of Pharmaceutical Sciences, University of Eastern Piedmont "Amedeo Avogadro", 28100 Novara, Italy; federica.pollastro@uniupo.it (Fe.P.);
* Correspondence: anrosa@unica.it

Table of Contents

Figure S1: Representative phase contrast images of SH-SY5Y cells after 24 h of incubation with the positive control gemcitabine.

Figure S2: Representative phase contrast images of SH-SY5Y cells after short-time incubation (2 h) with arzanol (ARZ), eupatilin (EUP), zerumbone (ZER), and xanthomicrol (XAN) and the positive control gemcitabine.

Figure S3: Linear relationships between arzanol (ARZ), eupatilin (EUP), zerumbone (ZER), and xanthomicrol (XAN) cytotoxic and apoptotic action (at 50 µM) and their computed properties reported in Table 3.

Figure S4:. ^{1}H NMR of xanthomicrol (XAN) in $CDCl_3$.

Figure S5: ^{1}H NMR of zerumbone (ZER) in $CDCl_3$.

Figure S6: ^{1}H NMR of eupatilin (EUP) in $CDCl_3$.

Figure S7: ^{1}H NMR of arzanol (ARZ) in $CDCl_3$.

 https://doi.org/10.3390/molecules30081742

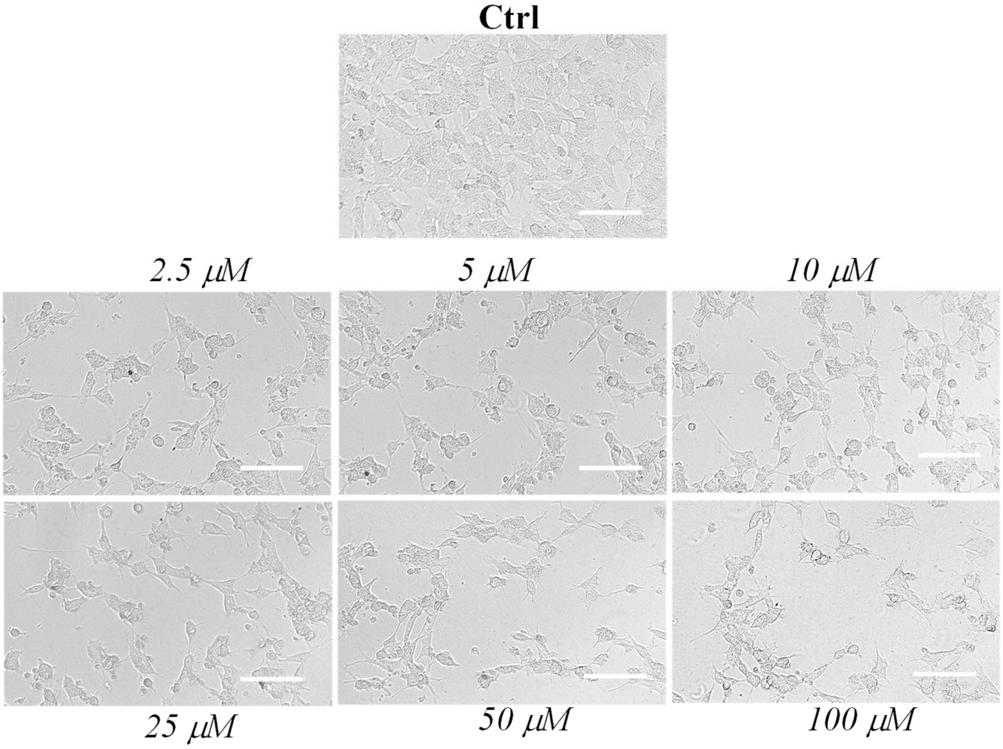

Figure S1. Representative phase contrast images of SH-SY5Y control cells (untreated, Ctrl) and cells treated for 24 h with different amounts (from 2.5 to 100 μM) of the anticancer compound gemcitabine (GEM, positive control). Bar = 100 μm.

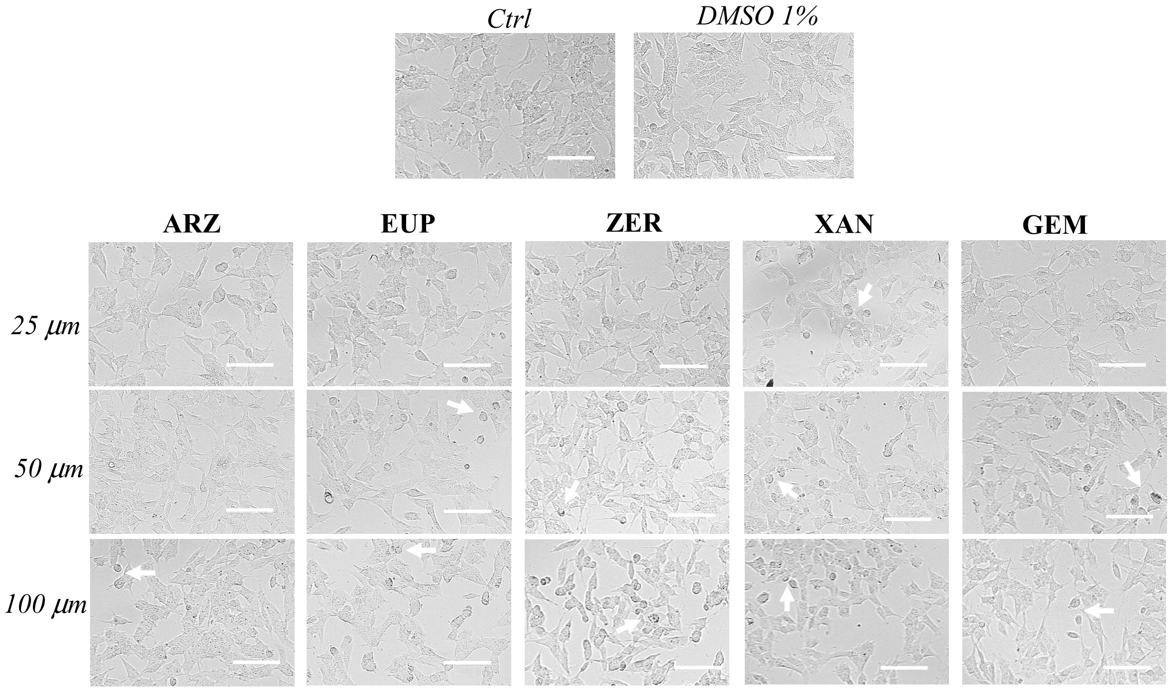

Figure S2. Representative phase contrast images of SH-SY5Y control cells (untreated, Ctrl) and cells treated for 2 h with different amounts (25, 50, and 100 µM) of arzanol (ARZ), eupatilin (EUP), zerumbone (ZER), xanthomicrol (XAN), and the anticancer compound gemcitabine (GEM, positive control). Arrows indicate rounded/granulated cells. Bar = 100 µm.

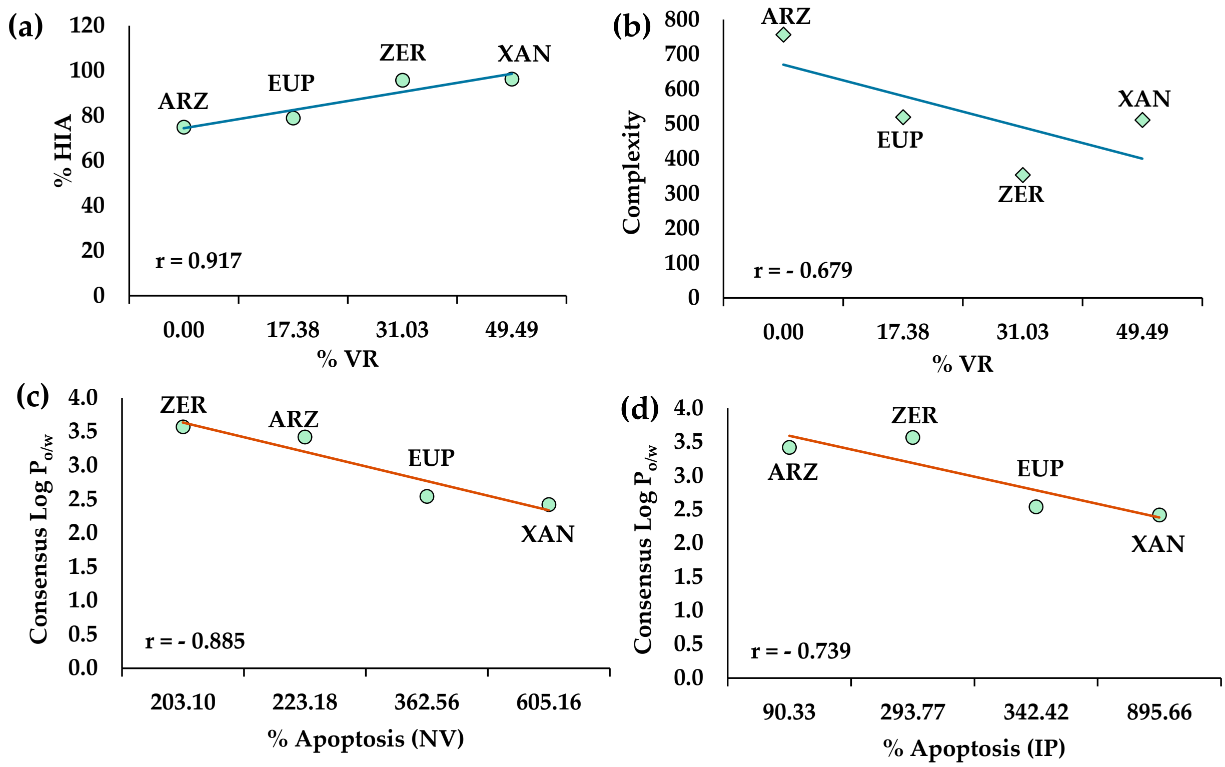

Figure S3. Linear correlation and Pearson's correlation coefficients (r) calculated for arzanol (ARZ), eupatilin (EUP), xanthomicrol (XAN), and zerumbone (ZER) at 50 µM between % viability reduction (% VR) and % human intestinal absorption (% HIA, Table 3) (**a**), % VR/complexity (**b**), % Apoptosis (NV)/Consensus Log $P_{O/W}$ (Table 3) (**c**), and % Apoptosis (IP)/Consensus Log $P_{O/W}$ (**d**).

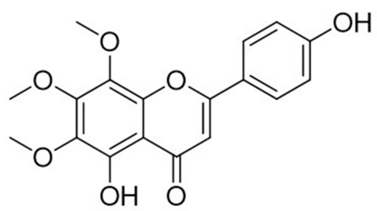

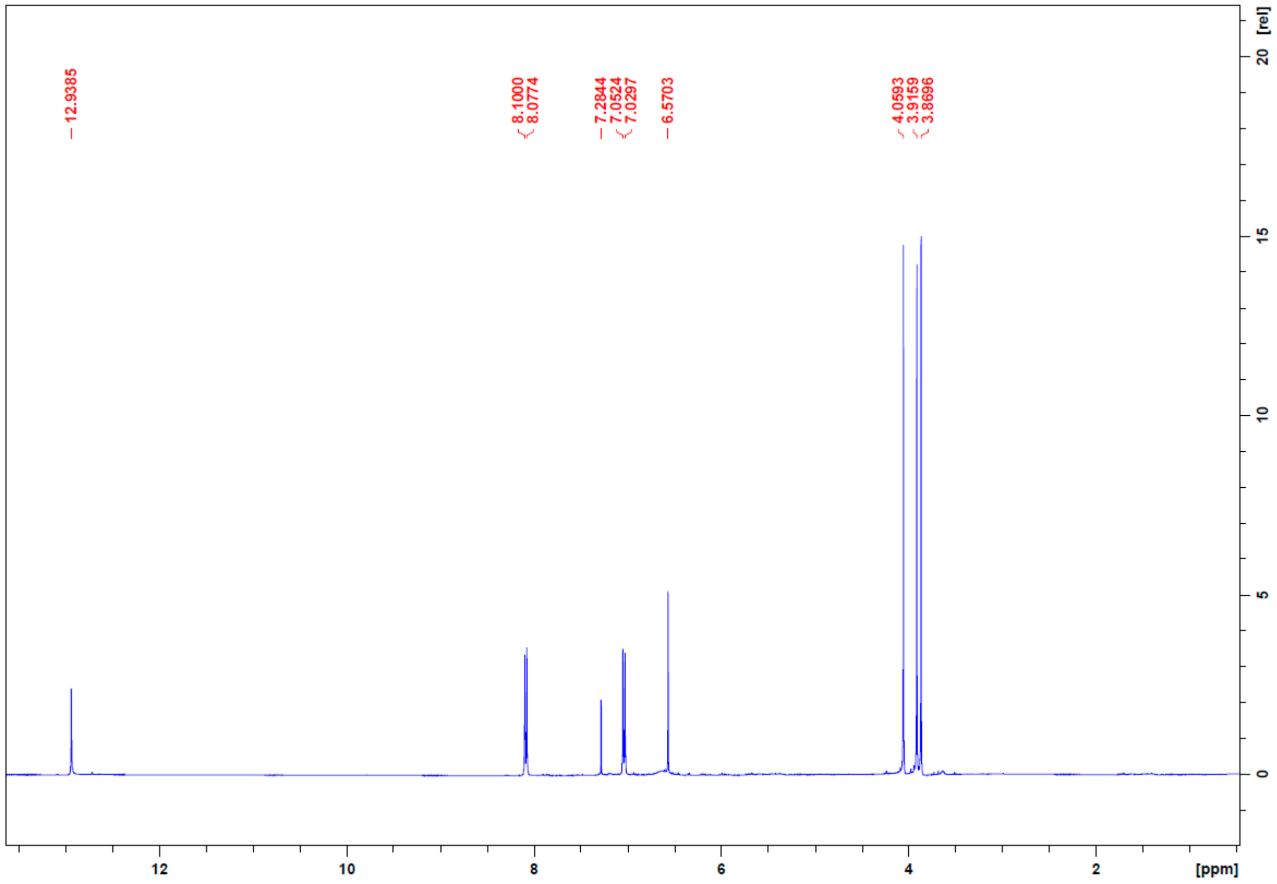

Figure S4. ^{1}H NMR of xanthomicrol (XAN) in $CDCl_3$.

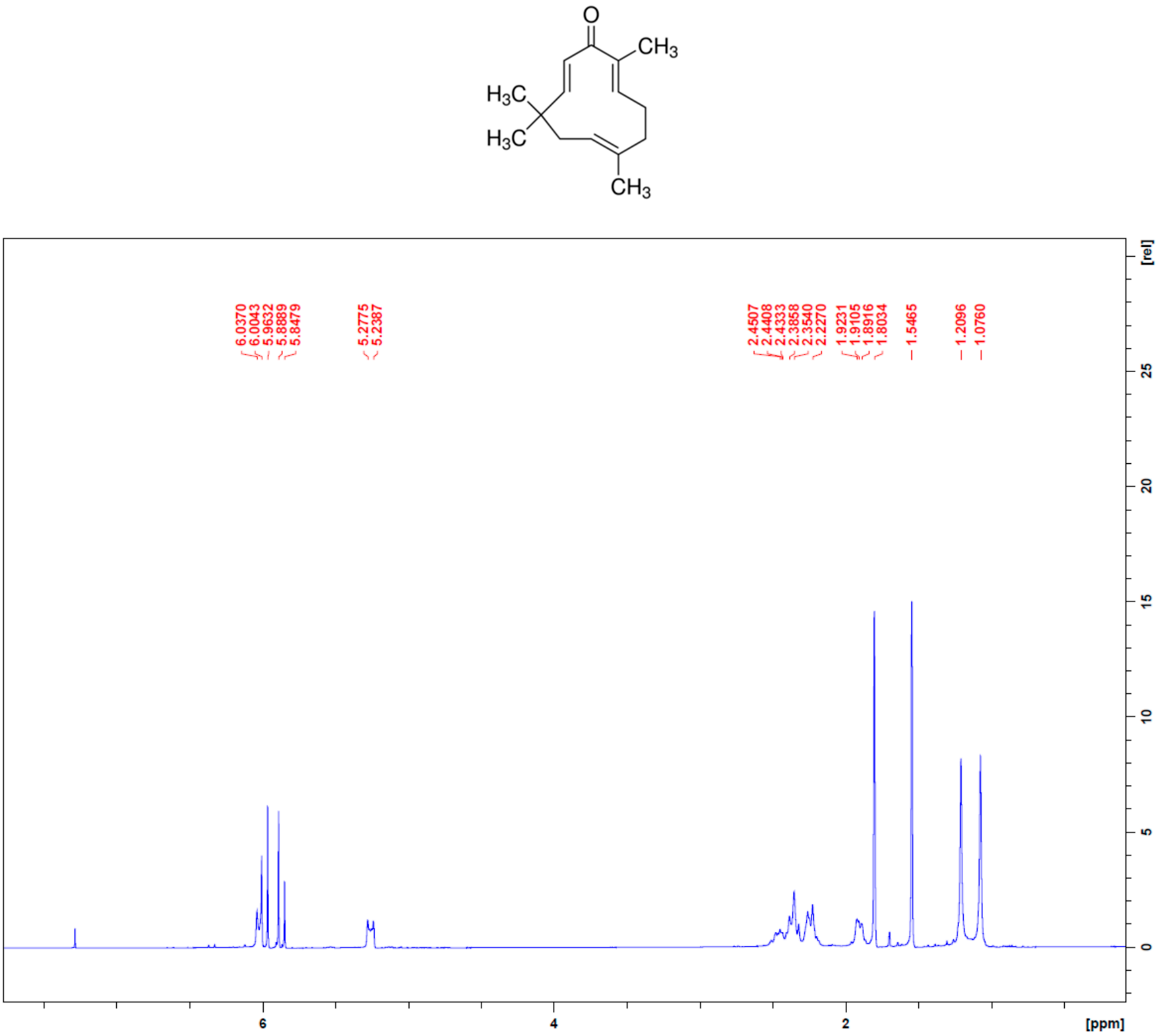

Figure S5. ^{1}H NMR of zerumbone in $CDCl_3$.

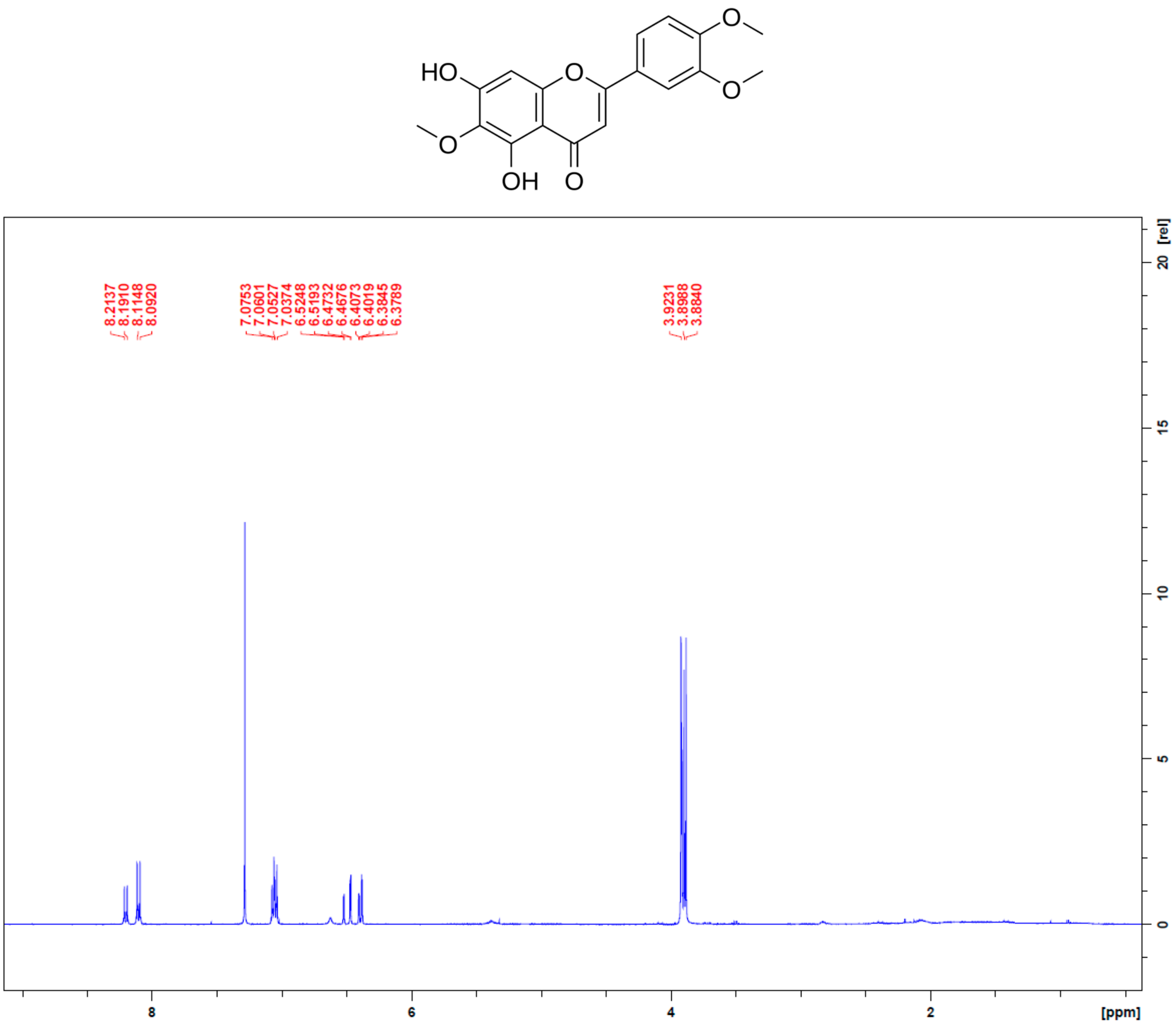

Figure S6. ^{1}H NMR of eupatilin in $CDCl_3$.

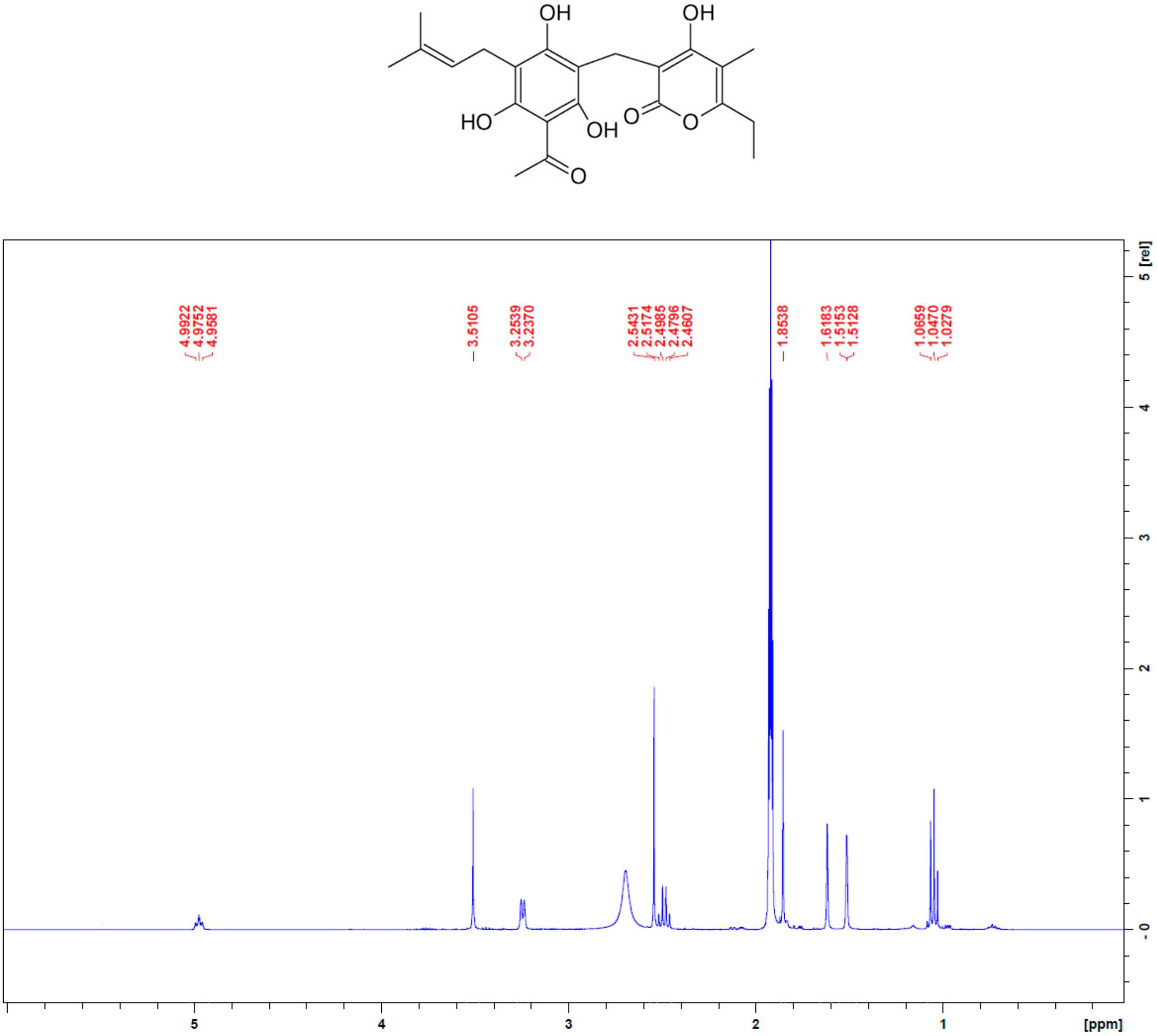

Figure S7. ^{1}H NMR of arzanol in $CDCl_3$.
